# Decoding the lipid-migraine link: a genetic and lipidomic investigation of migraine subtypes

**DOI:** 10.22514/jofph.2026.031

**Published:** 2026-05-12

**Authors:** Liang Zhang, Hong Li, Xiaocen Jin, Kaiyi Zhong, Lijun Liu, Jing Gao, Aijun Ma, Chunyan Jia

**Affiliations:** ^1^Department of Neurology, The Affiliated Hospital of Qingdao University, Qingdao University, 266071 Qingdao, Shandong, China; ^2^Department of Cardiology, The First Affiliated Hospital of Dalian Medical University, Dalian Medical University, 116011 Dalian, Liaoning, China

**Keywords:** Migraine, Mendelian randomization, Genome-wide association study, Lipids, Lipid species, Causal effect

## Abstract

**Background**: Migraine frequently co-occurs with cardiovascular and metabolic diseases. Observational studies examining the association between conventional lipid profiles and migraine risk have yielded inconsistent results and cannot establish causality. This study aimed to investigate the causal effects of specific lipid species on migraine and its primary subtypes: migraine with aura (MA) and without aura (MO). **Methods**: Using a Mendelian randomization (MR) methodology, this study analyzed genome-wide association study (GWAS) data from the UK Biobank and FinnGen Consortium. Exposures comprised seven lipids and 179 lipid species while the outcomes were overall migraine and its subtypes. Pleiotropy and heterogeneity were assessed using sensitivity analyses such as MR-Egger, weighted median, and Pleiotropy Residual Sum and Outlier (MR-PRESSO). **Results**: Genetically predicted higher levels of high-density lipoprotein cholesterol (HDL-C; odds ratio (OR) = 0.88; 95% confidence interval (CI), 0.82–0.93) and apolipoprotein A1 (ApoA1, OR = 0.89; 95% CI, 0.84–0.95) were associated with a reduced risk of migraine. Conversely, higher triglycerides (TG) increased the risk of overall migraine. Lipidomic analysis revealed 15 specific lipid species causally associated with overall migraine. Subtype-specific analyses revealed divergent causal profiles for MO and MA. Seven triacylglycerol (TAG) species were specifically associated with an increased risk of MO, whereas only sphingomyelins (SM) (d36:1) was linked to an increased risk of MA. **Conclusions**: This study provides robust evidence for a causal relationship between lipid metabolism and migraine, demonstrating that these effects are highly specific to individual lipid molecules and migraine subtypes. These findings enhance our understanding of the lipid-mediated mechanisms in migraine pathogenesis and highlight potential subtype-specific pathways for developing future therapeutic and preventive strategies.

## 1. Introduction

Migraine, a prevalent and complex neurological disorder affecting over 1 billion individuals globally and represents the second-leading greatest cause of disability-adjusted life years (DALYs) among neurological diseases [1, 2, 3]. Despite therapeutic advancements, effective prevention remains a significant challenge. This difficulty is compounded by well-established comorbidities with cardiovascular and metabolic diseases [4, 5, 6], as cohort studies report that migraine patients face a 1.5–2.5-fold higher risk of these conditions in migraine patients compared to the general population [7]. Dyslipidemia has been proposed as a key link within this comorbidity network. Observational studies have consistently associated abnormalities in conventional lipid profiles, such as elevated triglycerides (TG) and low-density lipoprotein cholesterol (LDL-C), with an increased risk and severity of migraine [8, 9, 10, 11]. However, the precise causal role of lipid metabolism in migraine pathogenesis remains unresolved. One proposed mechanism suggests that dyslipidemia may exacerbate systemic and neuroinflammation, thereby activating the trigeminovascular pathway [12]. Mendelian randomization (MR) studies have attempted to elucidate the causal nature of these associations. Recent meta-analyses and investigations have reported a protective effect for high-density lipoprotein cholesterol (HDL-C) and a risk-increasing effect for TG on migraine [13, 14, 15]. Although informative, these findings primarily reaffirm the role of aggregate lipid measures. This approach inherently limits biological interpretation, as composite lipids like total TG mask the contributions of specific lipid species that may have distinct and even opposing functions in neurological signaling and inflammation.

A critical, unaddressed question is whether the causal effects of lipids on migraine are mediated by specific molecular species and if these relationships differ between migraine with aura (MA) and without aura (MO), which are two subtypes with distinct pathophysiological profiles [16]. The structural and signaling functions of lipid subclasses, such as glycerophospholipids and sphingolipids, are highly relevant to migraine pathogenesis, as they regulate critical processes like neurotransmission (*e.g.*, serotonin, calcitonin gene-related peptide (CGRP)) and neuronal membrane stability [17, 18]. However, current evidence is derived almost exclusively from conventional lipid panels, leaving a significant knowledge gap in the understanding of lipidome- and subtype-specific mechanisms in migraine. Merely expanding the number of exposures in a Mendelian randomization analysis does not constitute a conceptual advance; the true novelty lies in disentangling the causal roles of individual lipid molecules across clinically distinct migraine subtypes.

We hypothesize that the causal relationship between dyslipidemia and migraine is not uniform but rather is driven by specific lipid species with subtype-specific effects. To test this hypothesis, we conducted a comprehensive MR analysis, using genetic variants as instrumental variables (IVs) to probe the causal links between an extensive panel of lipids and migraine. Departing from prior MR studies that were confined to broad lipid categories, our work is the first to systematically interrogate the causal effects of 179 distinct lipid species at the molecular level and to directly compare these effects between MA and MO. By integrating the latest genome-wide association study (GWAS) data from the UK Biobank and FinnGen Consortium, our study aims to map the causal landscape of the plasma lipidome onto migraine and its subtypes. This molecular-level approach represents a significant conceptual advance, moving beyond aggregated traits to pinpoint precise molecular players. We anticipate these findings will provide unprecedented detail into the lipid-related etiology of migraine, offering mechanistic insights to inform the development of subtype-specific biomarkers and tailored preventive strategies. Ultimately, this work seeks to bridge the critical gap between epidemiological association and actionable clinical understanding.

## 2. Materials and methods

### 2.1 Study design and flowchart

In our study, we implemented a two-sample Mendelian randomization (MR) framework to examine the causal effects of lipid traits (seven serum lipids and 179 lipid species) on migraine and its two primary subtypes: migraine without aura (MO) and migraine with aura (MA). A detailed flowchart outlining the core assumptions of MR and our specific analytical workflow is presented in Fig. 1. In this study, overall migraine and its sub-types were designated as the primary outcome, and the 179 lipid species constituted the primary exposure set. The causal associations between these primary exposures and the primary outcomes were considered the primary analysis. Associations tested in all other analyses, including those for seven conventional lipids, were considered exploratory.

**Fig. 1.** The flowchart of the Mendelian randomization study to explore causal associations between lipids and migraine. SNPs: single nucleotide polymorphisms; MR: Mendelian randomization; IVW: inverse-variance weighted.

### 2.2 Data source

The genome-wide association study (GWAS) data for plasma lipids were obtained from publicly available IEU OpenGWAS database (https://gwas.mrcieu.ac.uk/). Specifically, GWAS summary statistics for low-density lipoprotein cholesterol (LDL-C), high-density lipoprotein cholesterol (HDL-C), apolipoprotein B (ApoB), apolipoprotein A1 (ApoA1), and triglycerides (TG) were obtained from Richardson *et al*. [19]. Data for total cholesterol (TC) and lipoprotein(a) (Lp(a)) were sourced from Barton *et al*. [20], both utilizing the UK Biobank cohort. For lipidomic analyses, summary statistics from a large-scale plasma lipidome GWAS (https://www.ebi.ac.uk/gwas/, accession IDs GCST90277238–GCST90277416) were obtained via the GWAS Catalog. This dataset included 179 lipid species from the GeneRISK cohort (N = 7174 Finnish participants; 2595 males, 4579 females), categorized into four major classes: glycerolipids (GL), glycerophospholipids (GP), sphingolipids (SL), and sterols (ST). These classes further encompass 13 lipid subclasses: Triacylglycerols (TAG), diacylglycerols (DAG), lysophosphatidylcholines (LPC), lysophosphatidylethanolamines (LPE), phosphatidylcholines (PC), phosphatidylcholine ethers (PCO), phosphatidylethanolamines (PE), phosphatidylethanolamine ethers (PEO), phosphatidylinositols (PI), ceramides (Cer), sphingomyelins (SM), cholesteryl esters (CE)/sterol esters (SE), and cholesterol (Chol).

The effect estimates for all lipid exposures were scaled to a 1-standard deviation (SD) increase in their levels, which had undergone inverse normal transformation. Therefore, all reported odds ratios (ORs) represent the risk change per 1-SD increase in the respective lipid trait.

GWAS summary statistics for migraine were obtained from the FinnGen Consortium (R10 release, https://r10.finngen.fi/). Diagnoses were ascertained using International Classification of Diseases (ICD) codes, with ICD-10 as the primary system, supplemented by historical data mapped from ICD-9 and ICD-8. Overall migraine (G6_MIGRAINE) was defined by the ICD-10 code G43 (Migraine) or a relevant reimbursement code (R031 for prophylaxis or R032 for acute treatment). Migraine without Aura (MO) was defined as individuals with at least one specific diagnosis of G43.0 (Migraine without aura). To ensure the purity of the subtype analyses, the MO and MA groups were mutually exclusive. Individuals with a record of G43.1 (MA) or the unspecified code G43.9 were excluded from the MO group, and likewise, individuals with a record of G43.0 (MO) or G43.9 were excluded from the MA group. The final dataset comprised 20,908 cases and 312,803 controls for overall migraine (G6_MIGRAINE), 7593 cases and 312,803 controls for migraine without aura (G6_MIGRAINE_NO_AURA), and 8970 cases and 312,803 controls for migraine with aura (G6_MIGRAINE_WITH_AURA).

### 2.3 Instrumental variable selection

Instrumental variables (IVs) were selected to satisfy the three core MR assumptions: relevance, independence, and exclusion restriction (Fig. 1). First, to ensure relevance, we selected single nucleotide polymorphisms (SNPs) associated with each exposure at a genome-wide significance level (*p* < 5 × 10^−8^). While this stringent threshold was used for the seven conventional lipids, a more lenient threshold (*p* < 5 × 10^−6^) was applied for the 179 lipid species to ensure sufficient instrument strength for these less-studied traits. SNPs associated with migraine or its subtypes (*p* < 5 × 10^−8^) were excluded to minimize horizontal pleiotropy. Second, to ensure IV independence, we performed linkage disequilibrium (LD) clumping using an LD threshold of *r*^2^ < 0.001 within a 10,000 kb window. The robustness of IVs was assessed using the *F*-statistic and the proportion of variance explained (*R*^2^). To mitigate weak instrument bias, the strength of all IVs was assessed using the *F*-statistic, and any instrument with an *F*-statistic below 10 was excluded (**Supplementary Table 1**). Finally, we conducted two crucial harmonization and validation steps. Detailed metrics for all significant associations, including the number of SNPs (nSNP), mean *F*-statistic (with range), and *R*^2^ are provided in **Supplementary Tables 2,3,4**. Additionally, to confirm the correct direction of causality, we performed MR Steiger directionality tests for all significant associations. All reported findings passed this test (*p* < 0.05), confirming that the proportion of variance explained was greater in the exposure than in the outcome, which robustly supports the hypothesized causal direction (**Supplementary Table 1**). This stringent IV selection and validation process ensures the reliability of our causal inferences. Palindromic SNPs with ambiguous strand orientation or intermediate allele frequencies were removed to avoid directional mismatches [21]. Detailed IV metrics, including SNP counts, *F*-statistics, and *R*^2^, are provided in **Supplementary Tables 2,3,4**.

### 2.4 Statistical analysis

To explore genetic causal relationships between lipid traits and migraine, we applied five MR methods: MR-Egger regression, weighted median, inverse-variance weighted (IVW), simple mode and weighted mode, with the IVW method serving as the primary analysis due to its high statistical power. By default, a fixed-effects IVW model was used; however, in cases of significant heterogeneity (Cochran’s *Q* test *p* < 0.05), a random-effects IVW model was applied. To ensure the robustness of our findings and rigorously test for violations of MR assumptions, we conducted several sensitivity analyses. Specifically, directional pleiotropy was assessed via the MR-Egger intercept test (**Supplementary Table 5**), and heterogeneity among instrumental variables was quantified with Cochran’s *Q* statistic (**Supplementary Table 6**) and visually inspected using funnel plots. The Pleiotropy Residual Sum and Outlier (MR-PRESSO) test was used to detect and correct for outliers. Additionally, a leave-one-out analysis was performed to identify any single SNP driving the overall causal estimate. To account for multiple comparisons across the large number of traits tested, we controlled the false discovery rate (FDR) using the Benjamini-Hochberg procedure. An association was considered statistically significant only if its FDR-adjusted *p*-value was less than 0.05. Analyses were performed in R (version 4.4.1) using the TwoSampleMR package (v0.6.8) and MR-PRESSO package.

## 3. Result

### 3.1 Causal relationships between plasma lipoproteins and migraine

As illustrated in Fig. 2, our two-sample Mendelian randomization (MR) analysis revealed significant causal relationships between several conventional plasma lipids and the risk of migraine. Detailed genetic instrument information (**Supplementary Tables 7,8,9,10,11,12,13,14,15,16,17,18,19,20,21,22,23,24,25,26,27**) and scatter plots of MR effects (**Supplementary Figs. 1,2,3**) are provided. After correction for multiple testing, we found that genetically predicted higher levels of both high-density lipoprotein cholesterol (HDL-C) and apolipoprotein A1 (ApoA1) were robustly associated with a reduced risk of overall migraine (HDL-C: odds ratio based on the inverse-variance weighted method (ORIVW) = 0.88, 95% CI = 0.82–0.93, *p*-value adjusted by the false discovery rate (*p*_FDR_) <0.001; ApoA1: ORIVW = 0.89, 95% CI = 0.84–0.95, *p*_FDR_ = 0.001) (Fig. 2A). These protective associations were directionally consistent for both migraine without aura (MO) (Fig. 2B) and migraine with aura (MA) (Fig. 2C). Conversely, genetically predicted higher triglyceride (TG) levels were associated with an increased risk of both overall migraine (ORIVW = 1.10, 95% CI = 1.03–1.17, *p*_FDR_ = 0.009), and, more specifically, MO (ORIVW = 1.14, 95% CI = 1.04–1.25, *p*_FDR_ = 0.019). No significant association was observed between TG and MA. Although a nominal association was detected between Lp(a) and overall migraine risk, it did not survive correction for multiple testing (*p*_FDR_ = 0.072). Cochran’s *Q* test indicated heterogeneity for select associations (**Supplementary Table 6**). Random-effects IVW models, applied to heterogeneous results, yielded consistent estimates. Funnel plots demonstrated symmetry (**Supplementary Figs. 4,5,6**), and leave-one-out analyses confirmed no single SNP disproportionately influenced causal estimates (**Supplementary Figs. 7,8,9**). MR-Egger intercept tests and MR-PRESSO global tests revealed no evidence of horizontal pleiotropy (*p* > 0.05; **Supplementary Table 5**).

**Fig. 2.** The causal effect of seven lipids on migraine. Causal effect of seven lipids on (A) overall migraine, (B) migraine without aura and (C) migraine with aura using different methods of Mendelian randomization. ORs and 95% confidence intervals are presented per 1-SD increase in each lipid trait. nSNP: number of single nucleotide polymorphisms; OR: odds ratio; CI: confidence interval; FDR: false discovery rate; LDL-C: low-density lipoprotein cholesterol; HDL-C: high-density lipoprotein cholesterol; TG: triglycerides; TC: total cholesterol; ApoB: apolipoprotein B; ApoA1: apolipoprotein A1; MO: migraine without aura; MA: migraine with aura; *p*_FDR_: *p*-value adjusted by the false discovery rate.

### 3.2 Causal relationships between lipid species and migraine

To identify the specific molecular drivers of migraine, we analyzed causal associations between 179 lipid species and migraine risk. After FDR correction, we identified 15 individual lipid species with a significant causal effect on the risk of overall migraine (Fig. 3, **Supplementary Figs. 10,11**). Two species demonstrated protective effects including PC (16:0_16:0) (ORIVW = 0.91, 95% CI = 0.87–0.96, *p*_FDR_ = 0.025) and PCO (18:1_16:0) (ORIVW = 0.92, 95% CI = 0.87–0.97, *p*_FDR_ = 0.026) against migraine (Fig. 3, **Supplementary Figs. 10,11**). Thirteen lipid species were identified as risk factors for migraine (Fig. 3, **Supplementary Figs. 10,11**). These included DAG (18:1_18:2) (ORIVW = 1.07, 95% CI = 1.02–1.12, *p*_FDR_ = 0.034), PCO (18:1_20:3) (ORIVW = 1.09, 95% CI = 1.03–1.15, *p*_FDR_ = 0.034), SM (d36:1) (ORIVW = 1.09, 95% CI = 1.04–1.14, *p*_FDR_ = 0.025), SM (d38:1) (ORIVW = 1.06, 95% CI = 1.03–1.10, *p*_FDR_ = 0.025), TAG (48:1) (ORIVW = 1.14, 95% CI = 1.06–1.23, *p*_FDR_ = 0.025), TAG (48:2) (ORIVW = 1.11, 95% CI = 1.04–1.18, *p*_FDR_ = 0.027), TAG (51:3) (ORIVW = 1.09, 95% CI = 1.03–1.15, *p*_FDR_ = 0.027), TAG (52:4) (ORIVW = 1.08, 95% CI = 1.02–1.14, *p*_FDR_ = 0.034), TAG (53:2) (ORIVW = 1.10, 95% CI = 1.04–1.17, *p*_FDR_ = 0.025), TAG (53:3) (ORIVW = 1.08, 95% CI = 1.03–1.14, *p*_FDR_ = 0.025), TAG (54:4) (ORIVW = 1.09, 95% CI = 1.04–1.14, *p*_FDR_ = 0.025), TAG (54:5) (ORIVW = 1.12, 95% CI = 1.06–1.18, *p*_FDR_ = 0.008) and TAG (56:7) (ORIVW = 1.08, 95% CI = 1.03–1.13, *p*_FDR_ = 0.037).

**Fig. 3.** The causal effect of lipid species on migraine. The causal effect of fifteen lipid species on the risk of migraine. ORs and 95% confidence intervals are presented per 1-SD increase for lipid species. nSNP: number of single nucleotide polymorphisms; OR: odds ratio; CI: confidence interval; MR: Mendelian randomization; *p*_FDR_: *p*-value adjusted by the false discovery rate.

The robustness of these lipidomic-wide findings was corroborated by a comprehensive suite of sensitivity analyses. Cochran’s *Q* test revealed no significant heterogeneity across SNPs (*p* > 0.05; **Supplementary Table 6**). Funnel plots confirmed symmetry, supporting homogeneity (**Supplementary Fig. 12**). MR-Egger intercept tests found no evidence of horizontal pleiotropy (*p* > 0.05; **Supplementary Table 5**). Leave-one-out analyses confirmed that no single SNP disproportionately influenced the observed associations (**Supplementary Fig. 13**). Scatter plots illustrating causal effects between lipidomic species and migraine are provided in **Supplementary Fig. 11**.

### 3.3 Causal relationships between lipid species and migraine without aura (MO)

Our subtype-specific analysis for migraine without aura (MO) revealed a striking pattern: after FDR correction, all seven lipid species found to be causally associated with an increased risk of MO were triacylglycerols (TAGs) (Fig. 4, **Supplementary Figs. 14,15**). Five of these TAGs, TAG (51:3) (ORIVW = 1.15, 95% CI = 1.06–1.25, *p*_FDR_ = 0.034), TAG (53:2) (ORIVW = 1.19, 95% CI = 1.08–1.30, *p*_FDR_ = 0.022), TAG (53:3) (ORIVW = 1.15, 95% CI = 1.06–1.24, *p*_FDR_ = 0.022), TAG (54:4) (ORIVW = 1.14, 95% CI = 1.06–1.23, *p*_FDR_ = 0.024) and TAG (54:5) (ORIVW = 1.17, 95% CI = 1.07–1.27, *p*_FDR_ = 0.022) were also identified as risk factors for overall migraine (Fig. 4). Additionally, TAG (48:3) (ORIVW = 1.15, 95% CI = 1.05–1.26, *p*_FDR_ = 0.045) and TAG (52:3) (ORIVW = 1.15, 95% CI = 1.06–1.24, *p*_FDR_ = 0.022) were found to be specifically associated with an increased risk of MO (Fig. 4).

**Fig. 4.** The causal effect of lipid species on migraine without aura. The causal effect of seven lipid species on the risk of migraine without aura. ORs and 95% confidence intervals are presented per 1-SD increase for lipid species. nSNP: number of single nucleotide polymorphisms; OR: odds ratio; CI: confidence interval; MR: Mendelian randomization; *p*_FDR_: *p*-value adjusted by the false discovery rate.

Cochran’s *Q* test revealed no significant heterogeneity (*p* > 0.05; **Supplementary Table 6**). MR-Egger intercept tests detected no horizontal pleiotropy (*p* > 0.05; **Supplementary Table 5**). Funnel plots demonstrated symmetry (**Supplementary Fig. 16**) and leave-one-out analyses confirmed that no single SNP disproportionately influenced the causal estimates (**Supplementary Fig. 17**).

### 3.4 Causal relationships between lipid species and migraine with aura (MA)

In stark contrast to the findings for MO, our analysis for migraine with aura (MA) identified only a single lipid species with a significant causal effect after FDR correction. Genetically predicted higher levels of the sphingomyelin SM (d36:1) were associated with an increased risk of MA (ORIVW = 1.15, 95% CI = 1.07–1.23, *p*_FDR_ = 0.007) demonstrated a significant causal association with MA risk (Fig. 5, **Supplementary Figs. 18,19**). After heterogeneity and pleiotrophy analyses, no significant heterogeneity (**Supplementary Table 6** and **Supplementary Fig. 20**) and no horizontal pleiotropy (**Supplementary Table 5**) were determined. Leave-one-out analyses confirmed the robustness of the causal association (S**upplementary Fig. 21**).

**Fig. 5.** The causal effect of lipid species on migraine with aura. The causal effect of one lipid species on the risk of migraine without aura. ORs and 95% confidence intervals are presented per 1-SD increase for lipid species. nSNP: number of single nucleotide polymorphisms; OR: odds ratio; CI: confidence interval; MR: Mendelian randomization; *p*_FDR_: *p*-value adjusted by the false discovery rate.

## 4. Discussion

This Mendelian randomization (MR) study provides genetic evidence for the causal role of specific lipid profiles in migraine pathogenesis. Our findings confirm that genetically predicted higher levels of HDL-C and ApoA1 reduce the risk of overall migraine, whereas higher levels of triglycerides increase it; a nominal association for lipoprotein(a) (Lp(a)) did not withstand correction for multiple testing. Critically, our lipidomic-wide analysis demonstrates that these broad associations are underpinned by distinct molecular species with striking subtype specificity. We found that an increased risk of migraine without aura (MO) was driven by a cluster of several triacylglycerol (TAG) species. In stark contrast, the risk for migraine with aura (MA) was linked exclusively to a single sphingomyelin, SM (d36:1).

### 4.1 Interpreting Mendelian randomization evidence

Before discussing potential implications, it is critical to delineate the scope of inference from our MR design. Our study estimates the lifelong, population-level causal effect of genetic predisposition to altered plasma lipid levels on migraine risk. MR does not directly establish: (1) the consequences of acute, pharmacological lipid modification; (2) whether the observed effects are mediated by peripheral blood lipids, corresponding changes in the central nervous system, or systemic sequelae; or (3) the specific molecular pathways involved. The mechanistic discussions below are therefore presented as biologically plausible hypotheses intended to connect our causal evidence with existing neurobiology and to generate testable models for future research [22].

### 4.2 Conventional lipids and migraine pathophysiology

The comorbidity between migraine and cardiovascular disease is extensively documented [23, 24, 25, 26], and dyslipidemia may represent a key mechanistic bridge [11, 12, 27]. This link is likely mediated through shared pathological pathways, such as systemic inflammation and endothelial dysfunction, which are known to be central to migraine pathophysiology. Our results reinforce prior observations that ApoA1 and HDL-C are protective [13, 28]. This protective effect is likely mediated by the well-known anti-atherogenic and anti-inflammatory properties of these lipids [29, 30], which improve endothelial function and could thereby reduce migraine susceptibility. Mechanistically, HDL-C can downregulate endothelial adhesion molecules like intracellular cell adhesion molecule-1 (ICAM-1) and vascular cell adhesion molecule-1 (VCAM-1) [31, 32], both of which have been directly implicated in the inflammatory processes of migraine [33, 34].

Our analysis also provides insight into the roles of Lp(a) and triglycerides (TG). We identified a nominal positive association between genetically proxied Lp(a) and overall migraine risk; however, this finding did not withstand stringent correction for multiple testing and should thus be interpreted as suggestive. Should this association be validated in future studies, a plausible mechanism exists, as Lp(a) is a known promoter of atherosclerosis and endothelial dysfunction [35, 36], processes that can impair cerebral blood flow and plausibly influence migraine susceptibility [37, 38].

For TG, our MR analysis provides robust evidence for a causal, risk-increasing relationship with overall migraine and, more specifically, with MO, a finding that helps clarify previously inconsistent results from observational studies [9, 14, 15, 39]. This conclusion is powerfully substantiated at the molecular level by our lipidomic data, which revealed that a distinct cluster of TAG species specifically elevated the risk of MO. A potential mechanism unifying these findings is that hypertriglyceridemia may exacerbate the pro-inflammatory state and enhance platelet activity, both of which are well-established processes in migraine pathophysiology [40, 41].

### 4.3 Lipidomic insights and hypotheses for subtype-specific mechanisms

The novelty of our study lies in its ability to move beyond aggregate lipids and propose mechanisms for specific molecular species. PCs and PEs constitute the most abundant glycerophospholipids in eukaryotic cell membranes [42, 43]. The protective effect of the phosphatidylcholine PC (16:0_16:0), also known as dipalmitoyl phosphatidylcholine (DPPC), likely relates to its biophysical properties. As a fully saturated phospholipid, DPPC is known to increase the stability and order of cell membranes [44, 45]. We therefore hypothesize that its enrichment could stabilize neuronal membranes, thereby increasing the threshold for neuronal hyperexcitability and cortical spreading depression (CSD), and modulating the function of pain-signaling receptors housed within membrane microdomains [46].

Our finding of opposing causal effects for two ether-linked phosphatidylcholines (PCOs) offers a compelling mechanistic clue. While PCO (18:1_16:0) was protective, PCO (18:1_20:3) was risk-increasing. The critical difference lies in their sn-2 fatty acid: the former contains palmitic acid (16:0), while the latter contains dihomo-γ-linolenic acid (20:3, n-6), a direct precursor to arachidonic acid and pro-inflammatory eicosanoids like prostaglandin E2 (PGE2) [47], which is a well-established migraine trigger [48]. This divergence strongly suggests that the sn-2 fatty acid composition of ether lipids is a key determinant of their effect on migraine, potentially by modulating the balance between pro- and anti-inflammatory signaling pools [49].

Our observation that the sphingomyelin SM (d36:1) was exclusively linked to an increased risk of MA points toward a mechanism central to the aura phenotype. As key components of membrane microdomains [50], sphingolipids and their metabolites, such as ceramide and sphingosine-1-phosphate (S1P) [51], are deeply involved in neuroinflammation and nociceptive signaling. Given that CSD is the neurobiological correlate of aura, we propose that elevated SM (d36:1) may alter the properties of these microdomains in a way that lowers the threshold for CSD initiation or propagation [52], a possibility that requires direct experimental testing.

The causal link between the diacylglycerol DAG (18:1_18:2) and increased migraine risk suggests a potential dual-hit mechanism. As a key second messenger [53], DAG directly activates protein kinase C (PKC), which can promote neuronal hyperexcitability. Concurrently, its sn-2 linoleic acid component can be metabolized into pro-inflammatory substrates [54], offering a second pathway through which this lipid species may increase migraine susceptibility.

### 4.4 Strengths and limitations

This study has notable strengths, including the use of updated, large-scale GWAS data with refined phenotyping, and a detailed lipidomic MR approach that moves beyond broad lipid classes. This molecular-level approach enabled us to move beyond conventional lipid panels to delineate distinct, subtype-specific causal relationships for migraine, thereby generating targeted hypotheses for future translational research.

Certain limitations, however, warrant consideration. First, our reliance on GWAS data from individuals of predominantly European ancestry means that these findings require validation in more diverse populations to establish generalizability. Second, while we employed robust methods to mitigate bias from sample overlap, the potential for shared participants between the Finnish exposure (GeneRISK) and outcome (FinnGen) cohorts cannot be entirely excluded. Third, data constraints prevented important subgroup analyses stratified by age, sex, or body mass index (BMI), and we were unable to adjust for the potential confounding effects of lipid-lowering medications. Fourth, it is crucial to reiterate that while MR provides strong evidence for causality, it does not elucidate the specific biological pathways involved, and our use of lipid measurements from a single time-point does not capture the dynamic nature of the plasma lipidome.

## 5. Conclusions

In conclusion, this large-scale Mendelian randomization study provides robust genetic evidence supporting causal roles for specific lipid profiles in migraine, with distinct effects for MO and MA. The transition from conventional lipids to specific lipid species offers a refined view of this relationship. While our MR design establishes correlation consistent with causality, the specific biological mechanisms require further experimental investigation. Collectively, these findings deepen our understanding of the lipid-migraine nexus and identify novel, subtype-specific targets for the development of future preventive and therapeutic strategies.

## Supplementary Material

Supplementary material associated with this article can be found, in the online version, at https://files.jofph.com/ files/article/2043883583980879872/attachment/ Supplementary%20material.zip.




