# Supplementary material for: Decoding the lipid-migraine link: a genetic and lipidomic investigation of migraine subtypes
Source: J Oral Facial Pain Headache. 2026 May 12;40(3):156–66. doi: 10.22514/jofph.2026.031 (PMC13223921; doi:10.22514/jofph.2026.031)
Supplement: Supplementary file 1 [file Supplementary-Figures.pdf]

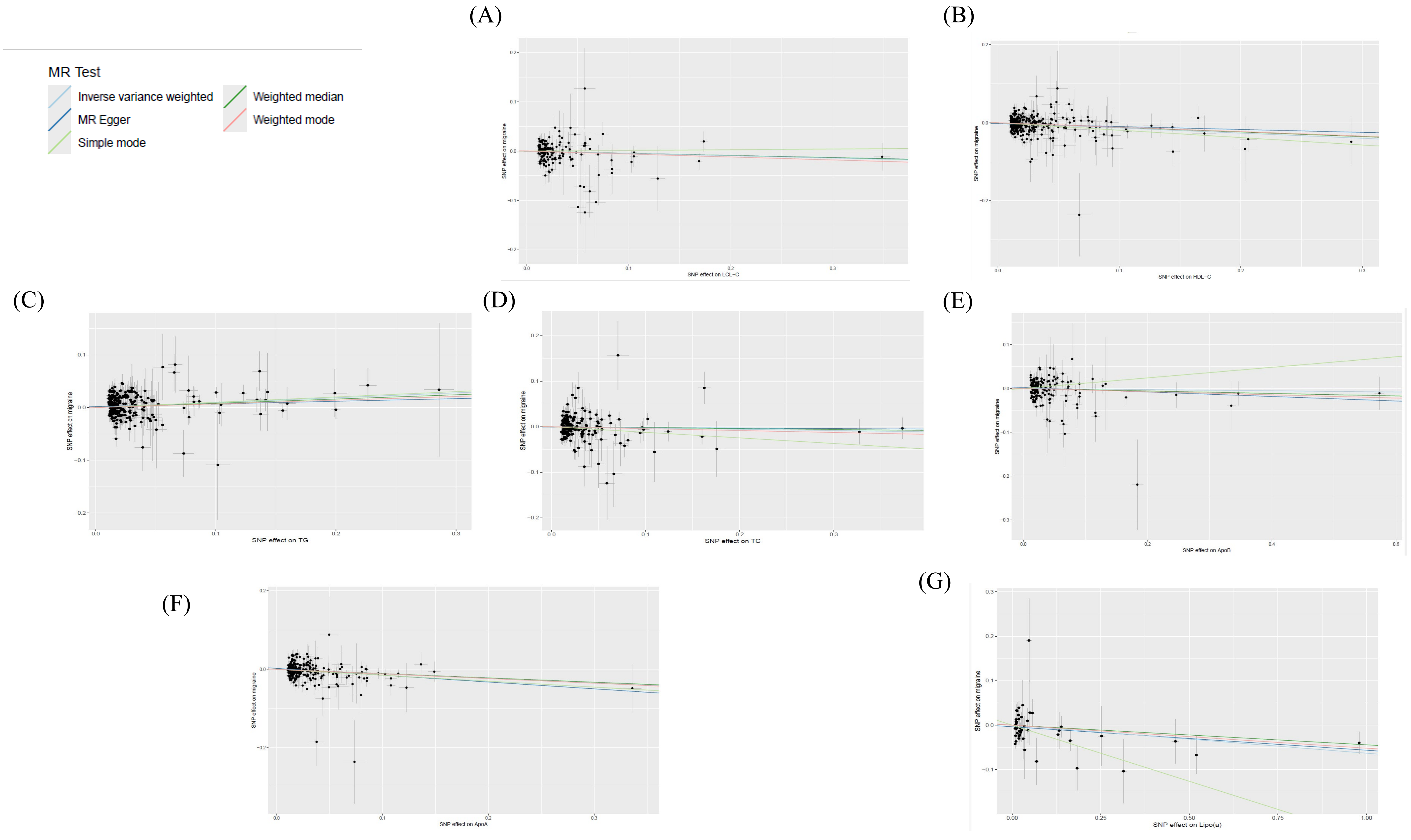

Supplementary Fig. 1. Scatter plots to show MR estimates of (A) LDL-C, (B) HDL-C, (C) TG, (D) TC, (E) ApoB, (F) ApoA1 and (G) lipoprotein (a) on migraine.

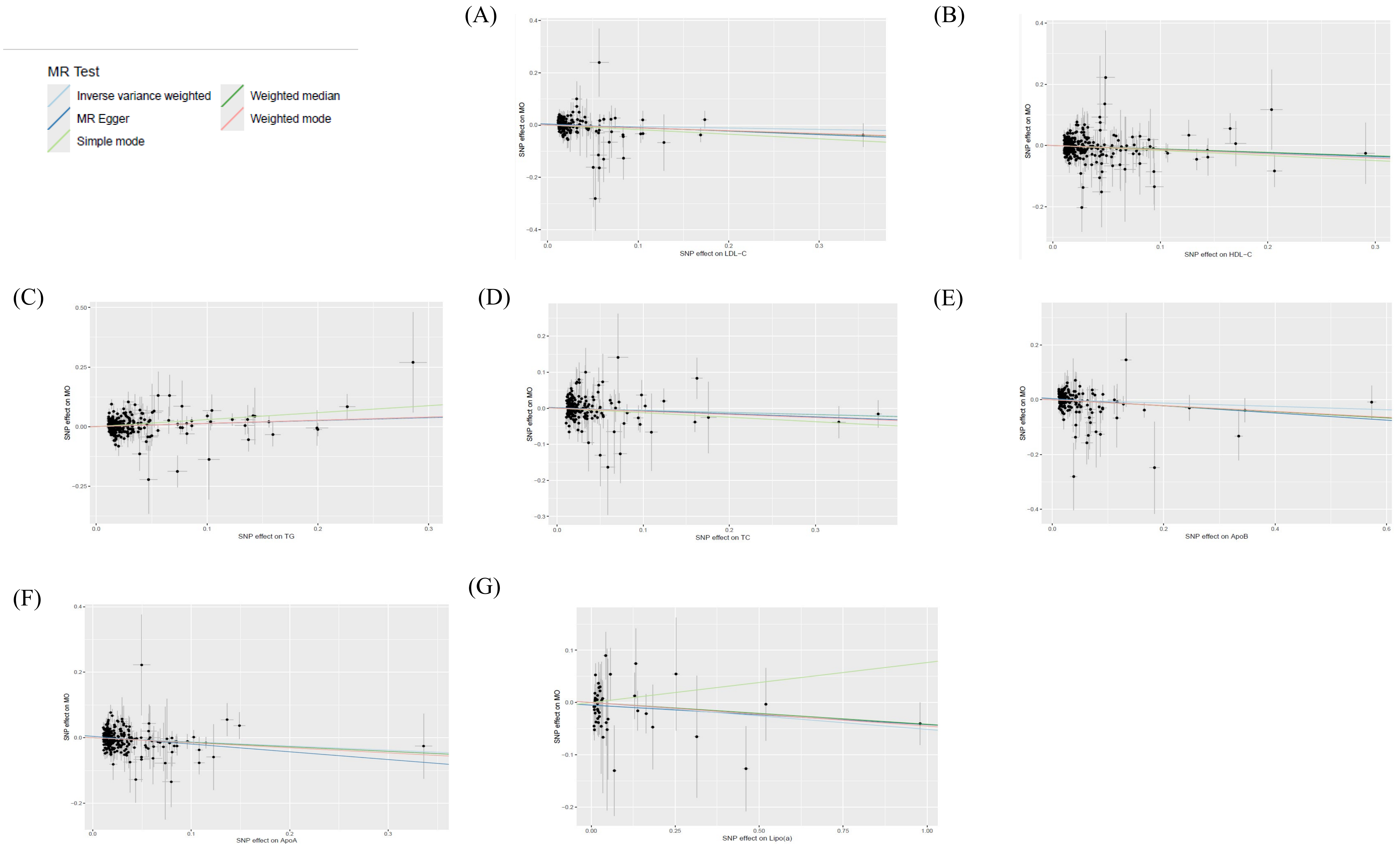

Supplementary Fig. 2. Scatter plots to show MR estimates of (A) LDL-C, (B) HDL-C, (C) TG, (D) TC, (E) ApoB, (F) ApoA1 and (G) lipoprotein (a) on migraine without aura (MO).

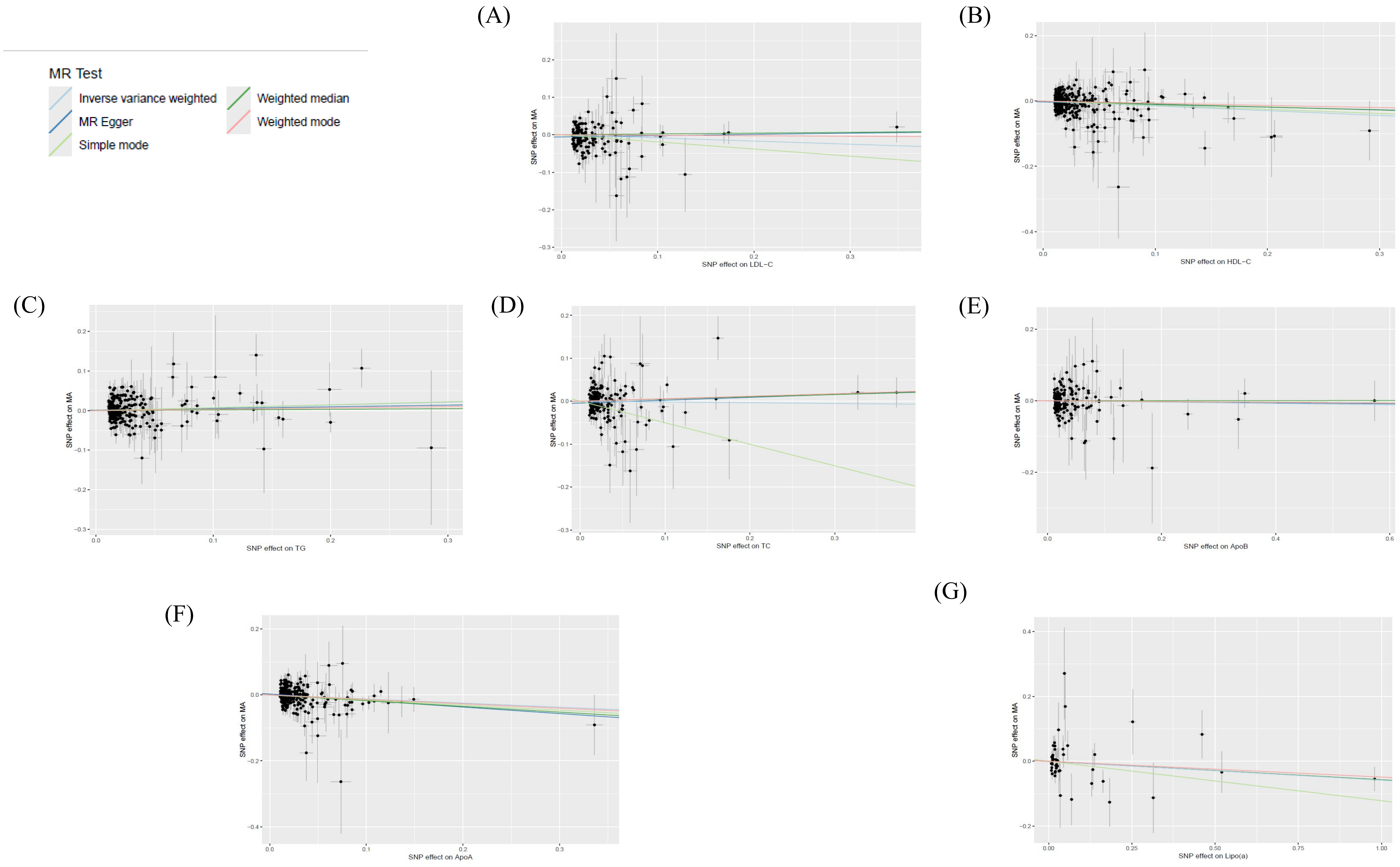

Supplementary Fig. 3. Scatter plots to show MR estimates of (A) LDL-C, (B) HDL-C, (C) TG, (D) TC, (E) ApoB, (F) ApoA1 and (G) lipoprotein (a) on migraine with aura (MA).

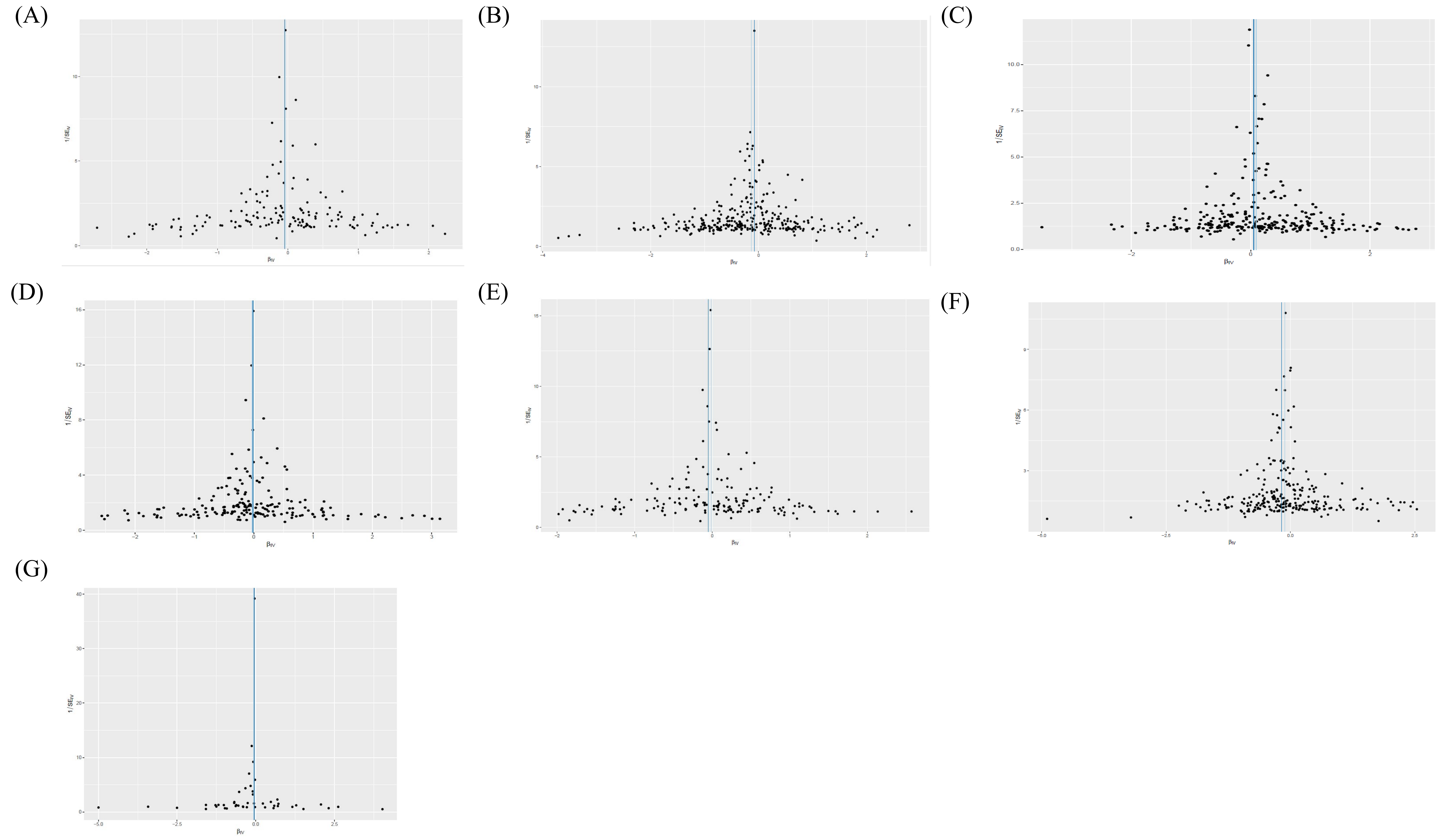

Supplementary Fig. 4. Funnel plots of MR analyses of (A) LDL-C, (B) HDL-C, (C) TG, (D) TC, (E) ApoB, (F) ApoA1 and (G) lipoprotein (a) on migraine.

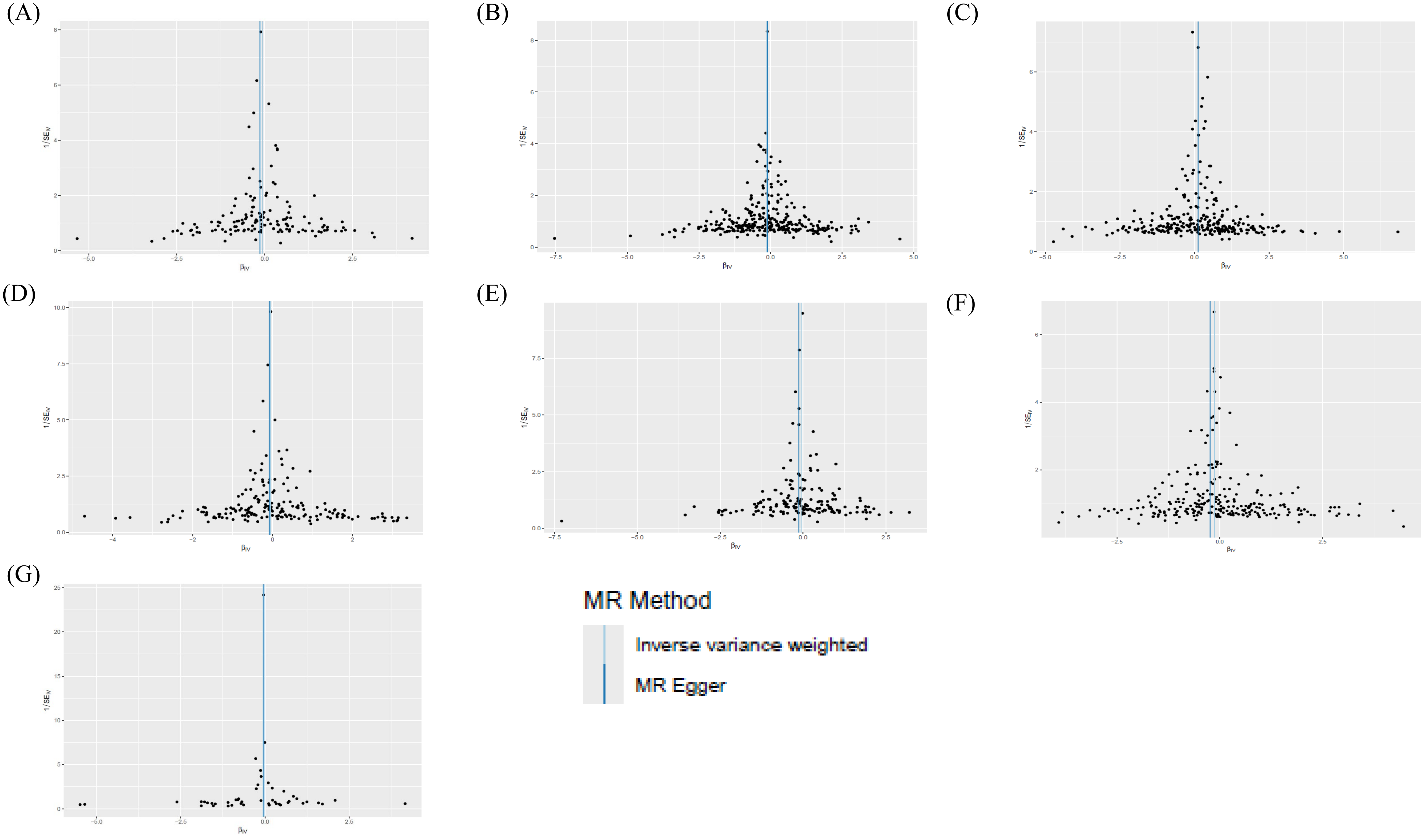

Supplementary Fig. 5. Funnel plots of MR analyses of (A) LDL-C, (B) HDL-C, (C) TG, (D) TC, (E) ApoB, (F) ApoA1 and (G) lipoprotein (a) on migraine without aura (MO).

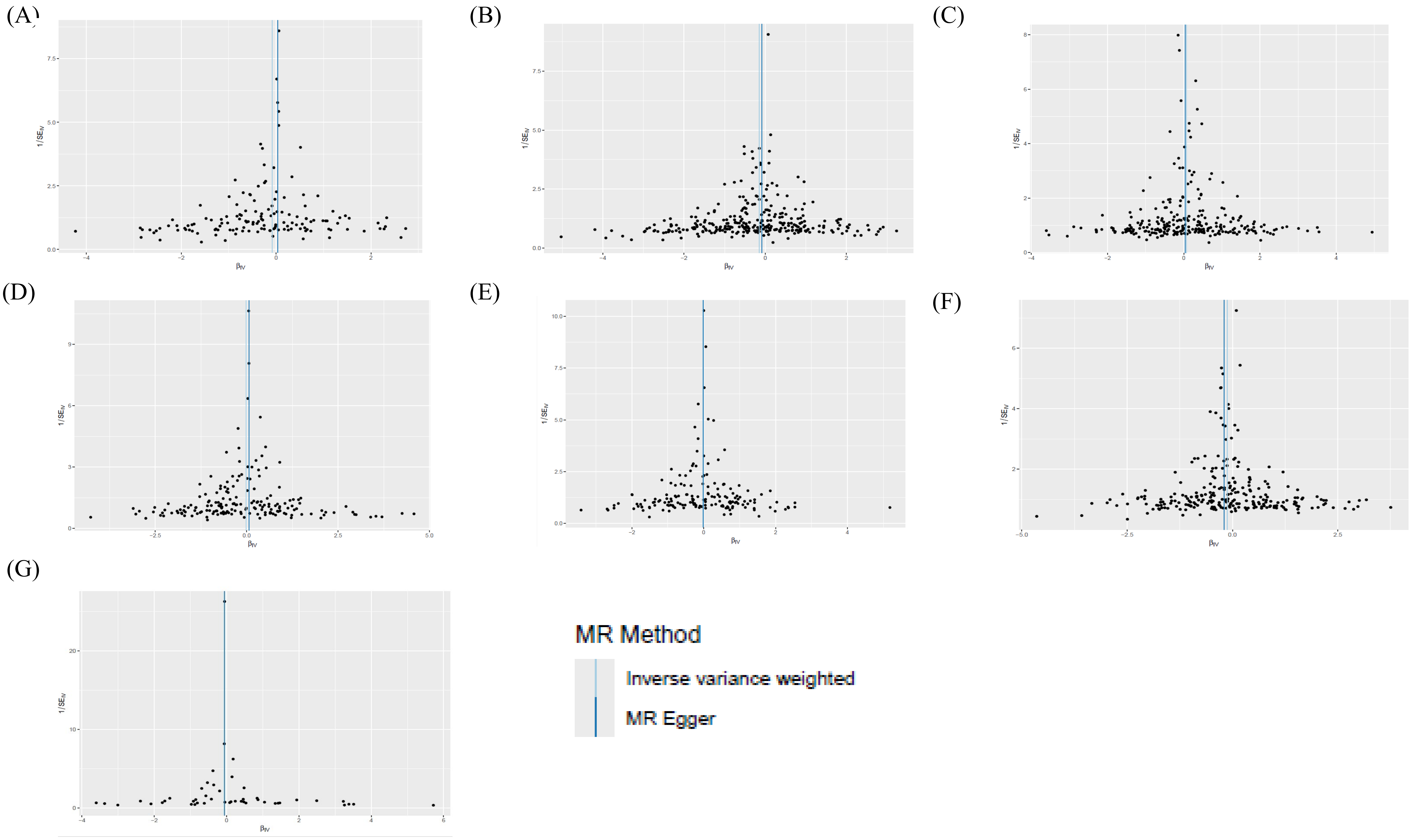

Supplementary Fig. 6. Funnel plots of MR analyses of (A) LDL-C, (B) HDL-C, (C) TG, (D) TC, (E) ApoB, (F) ApoA1 and (G) lipoprotein (a) on migraine migraine with aura (MA).

(A)

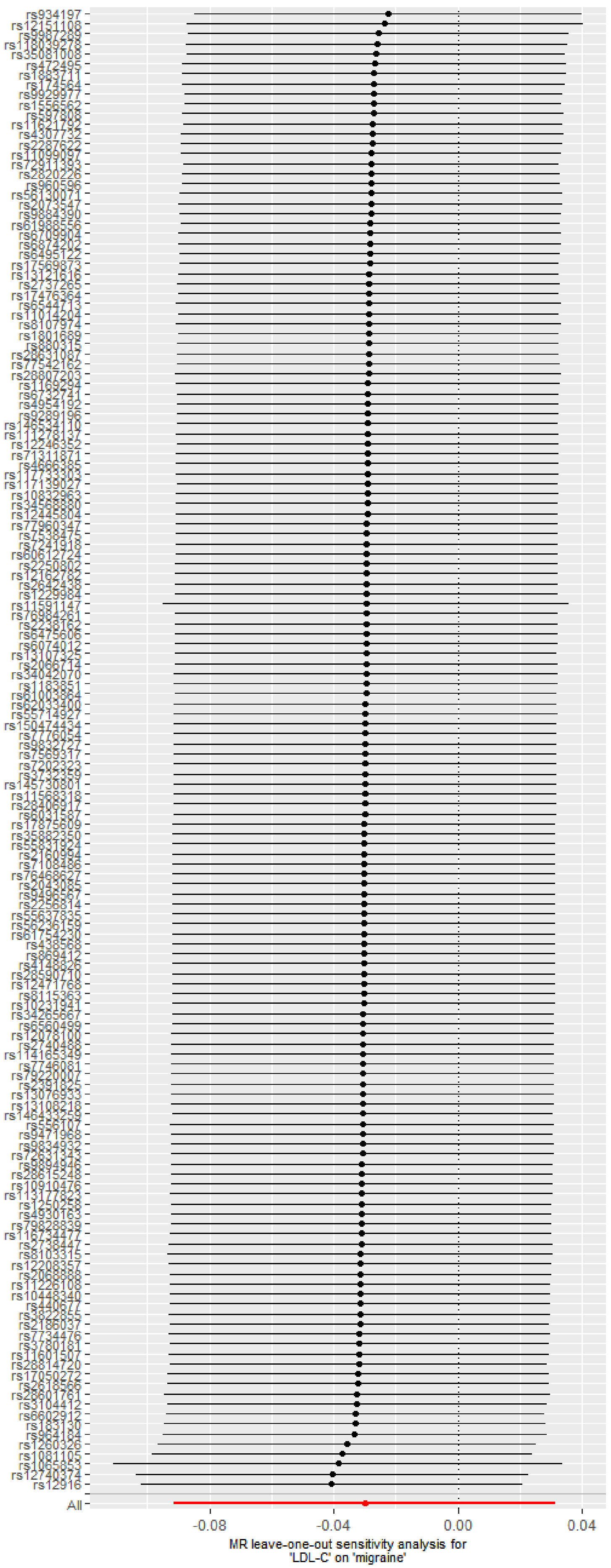
All
-0.08
-0.04
0.00
0.04
MR leave-one-out sensitivity analysis for
'LDL-C' on 'migraine'

(B)

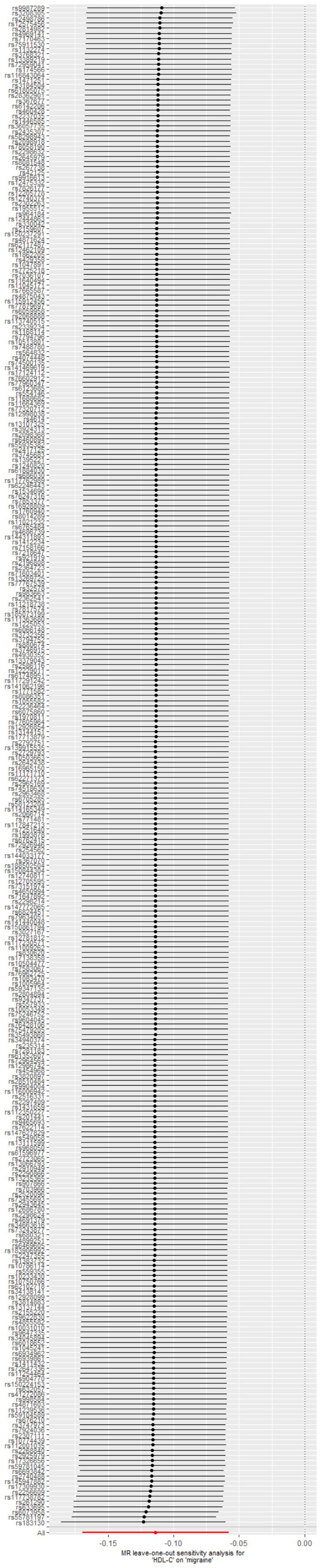
All
-0.15
-0.10
-0.05
0.00
MR leave-one-out sensitivity analysis for
'HDL-C' on 'migraine'

(C)

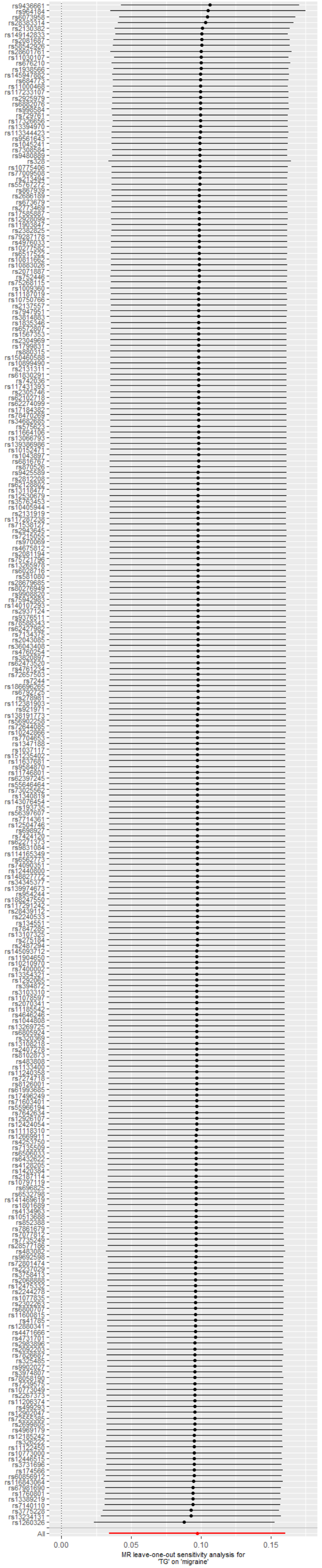
All
0.00
0.05
0.10
0.15
MR leave-one-out sensitivity analysis for
'TG' on 'migraine'

(D)

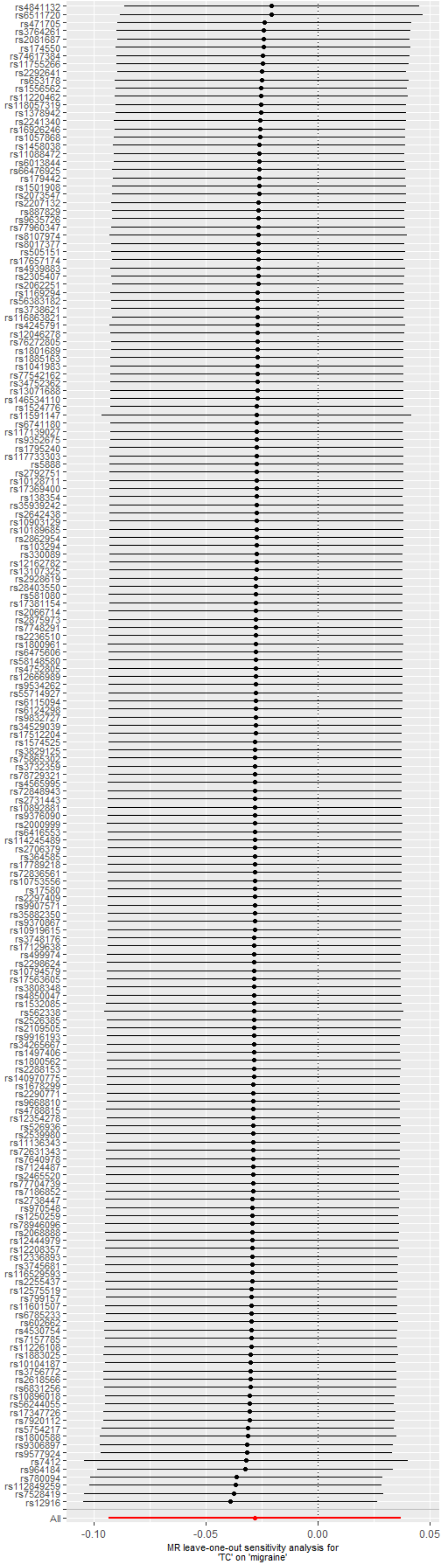
All
-0.10
-0.05
0.00
0.05
MR leave-one-out sensitivity analysis for
'TC' on 'migraine'

(E)

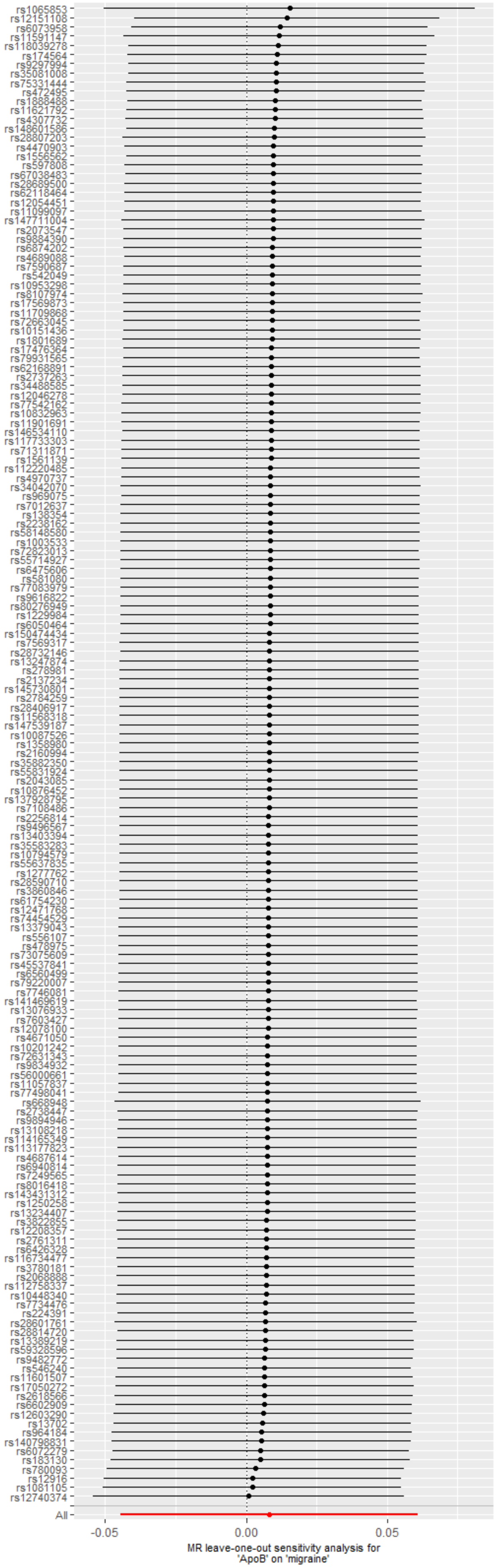

All
-0.05
0.00
0.05
MR leave-one-out sensitivity analysis for
'ApoB' on 'migraine'

(F)

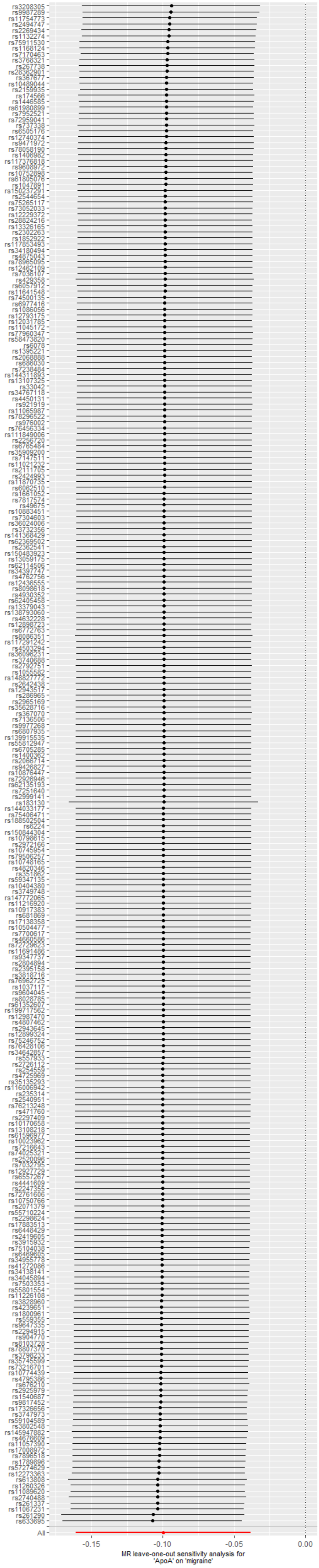

All
-0.15
-0.10
-0.05
0.00
MR leave-one-out sensitivity analysis for
'ApoA' on 'migraine'

(G)

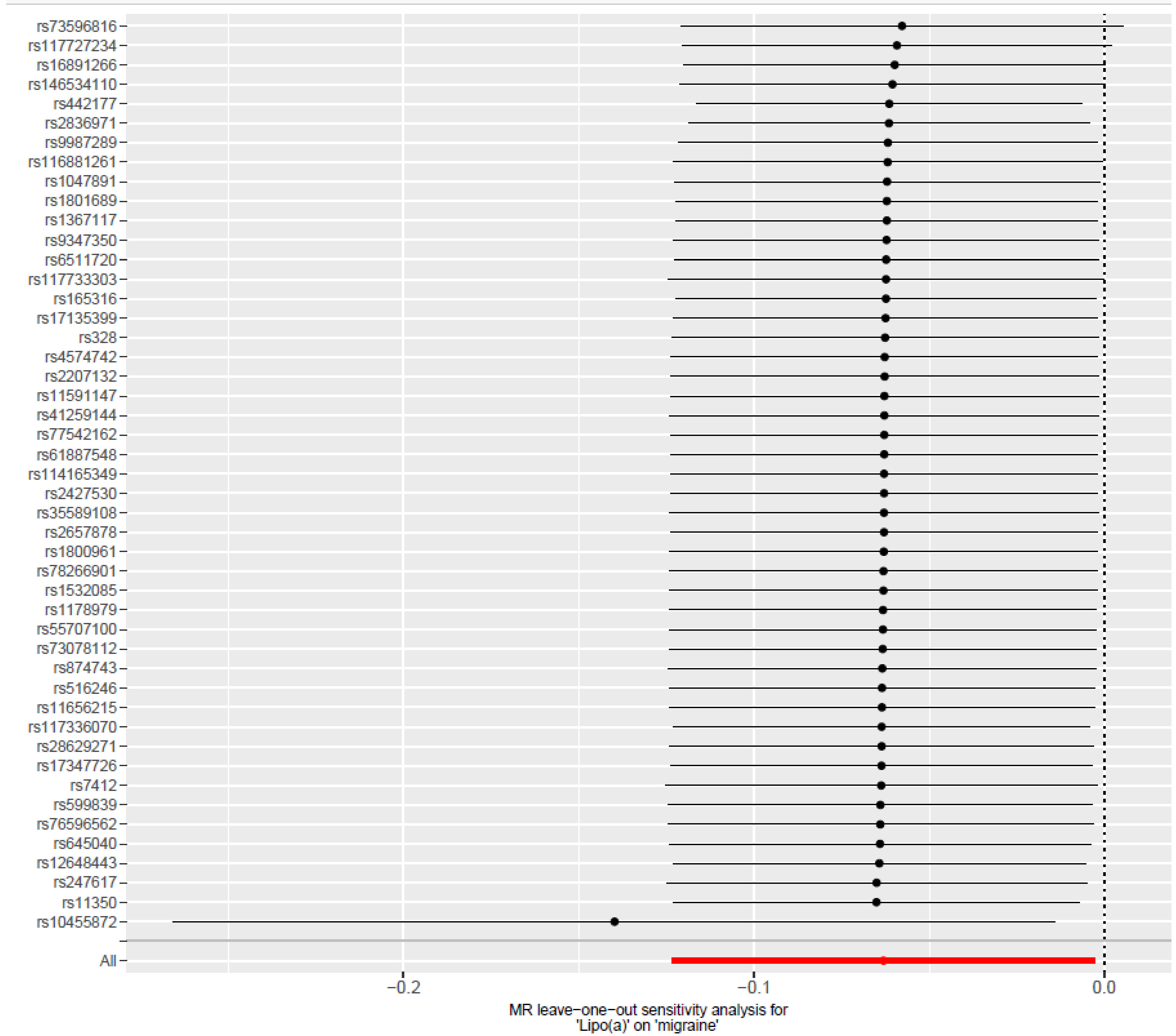

Supplementary Fig. 7. Leave-one-out analysis of MR analyses of (A) LDL-C, (B) HDL-C, (C) TG, (D) TC, (E) ApoB, (F) ApoA1 and (G) lipoprotein (a) on migraine.

(A)

(B)

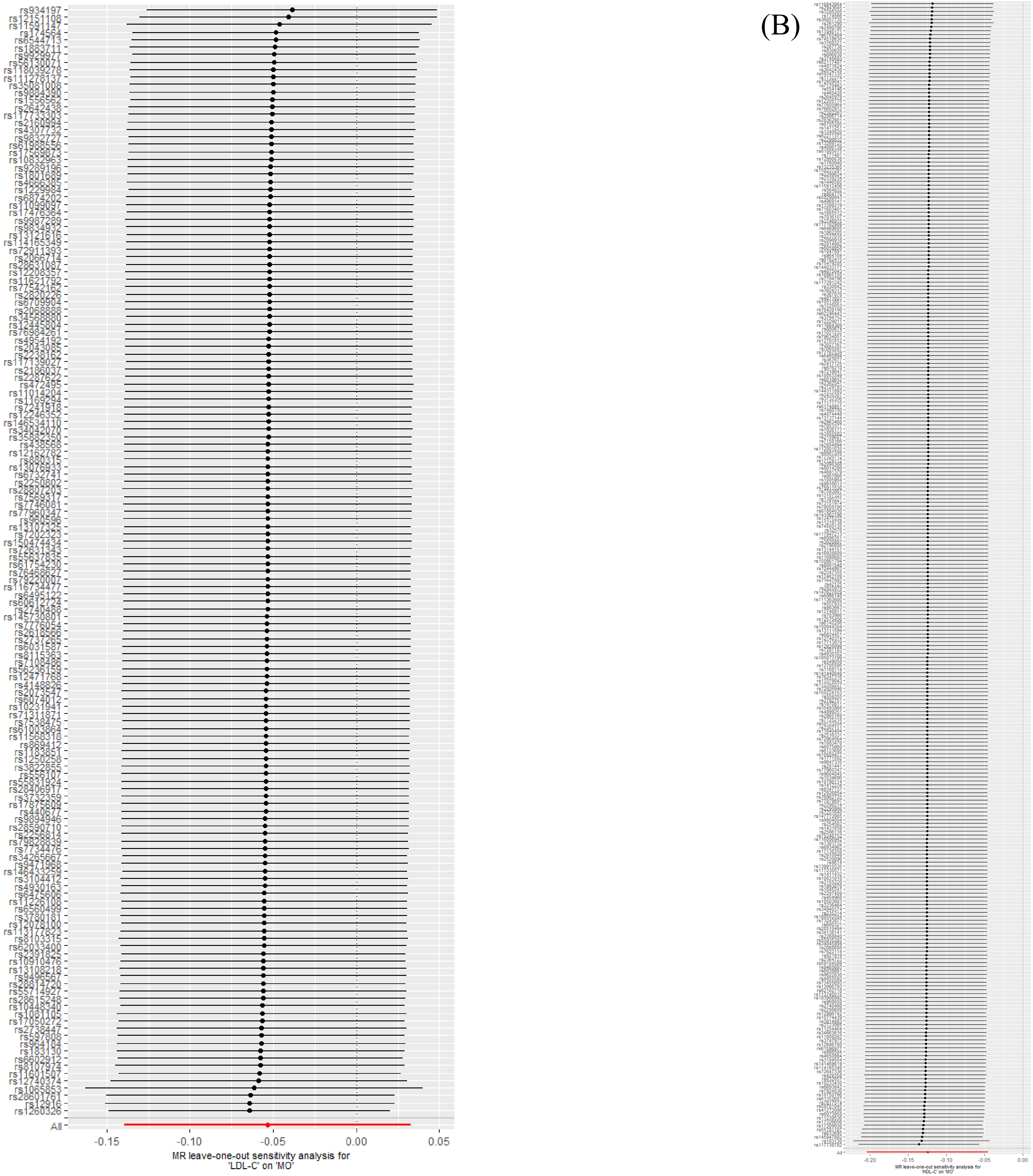
All
-0.15
-0.10
-0.05
0.00
0.05
MR leave-one-out sensitivity analysis for
'LDL-C' on 'MO'
-0.20
-0.15
-0.10
-0.05
0.00
MR leave-one-out sensitivity analysis for
'HDL-C' on 'MO'

(C)

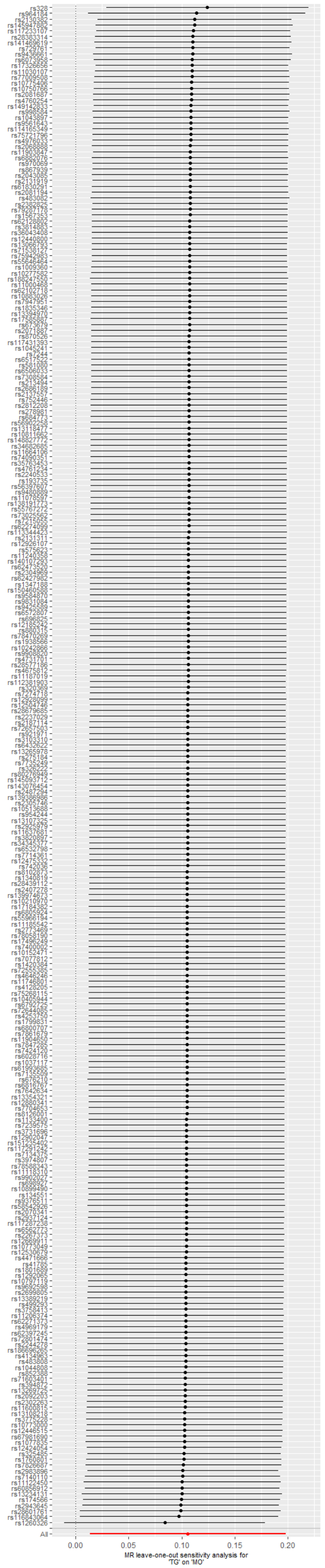
0.00
0.05
0.10
0.15
0.20
MR leave-one-out sensitivity analysis for
'TG' on 'MO'

(D)

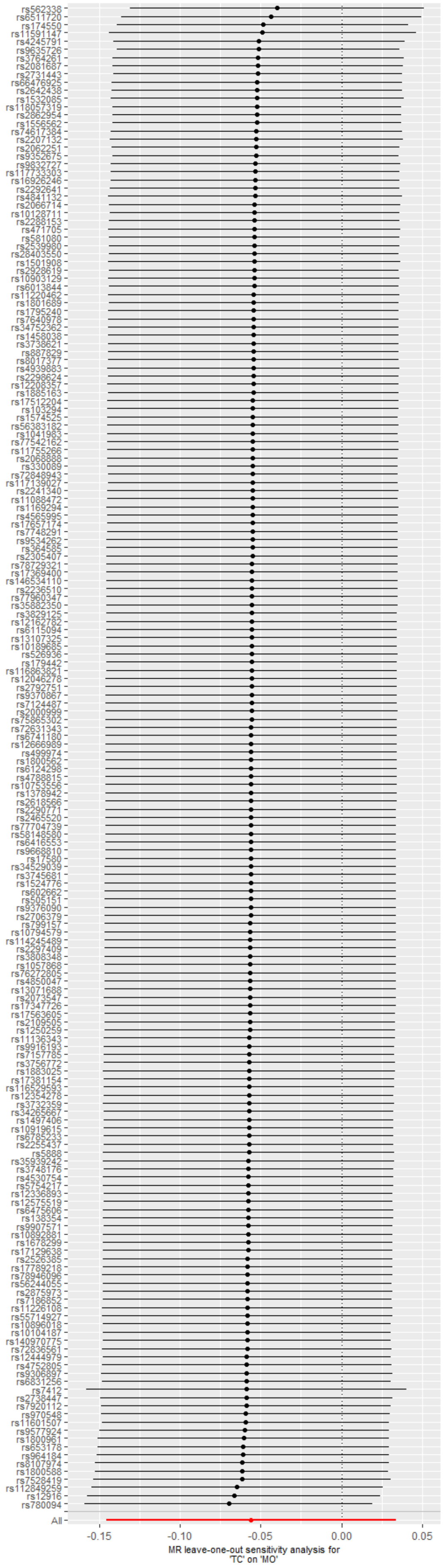
-0.15
-0.10
-0.05
0.00
0.05
MR leave-one-out sensitivity analysis for
'TC' on 'MO'

(E)

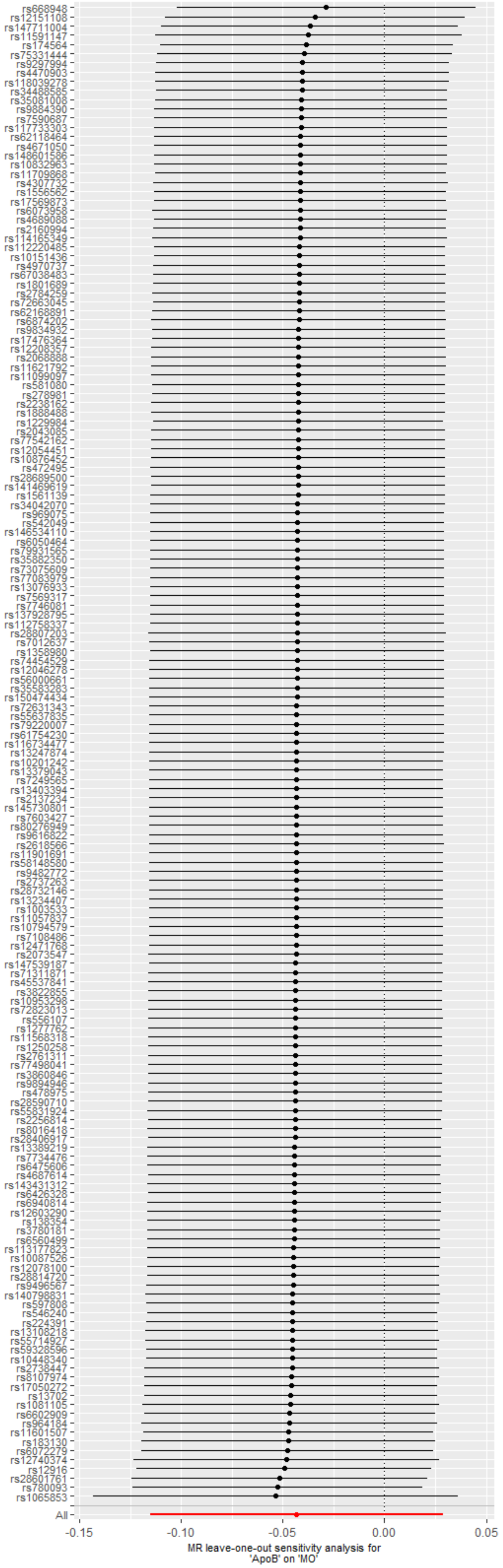
All
-0.15
-0.10
-0.05
0.00
0.05
MR leave-one-out sensitivity analysis for
'ApoB' on 'MO'

(F)

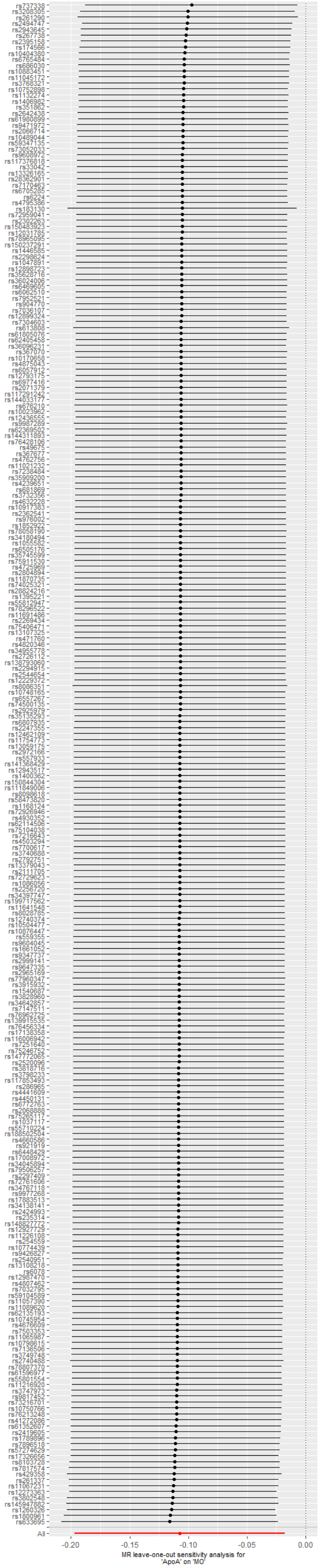
All
-0.20
-0.15
-0.10
-0.05
0.00
MR leave-one-out sensitivity analysis for
'ApoA' on 'MO'

(G)

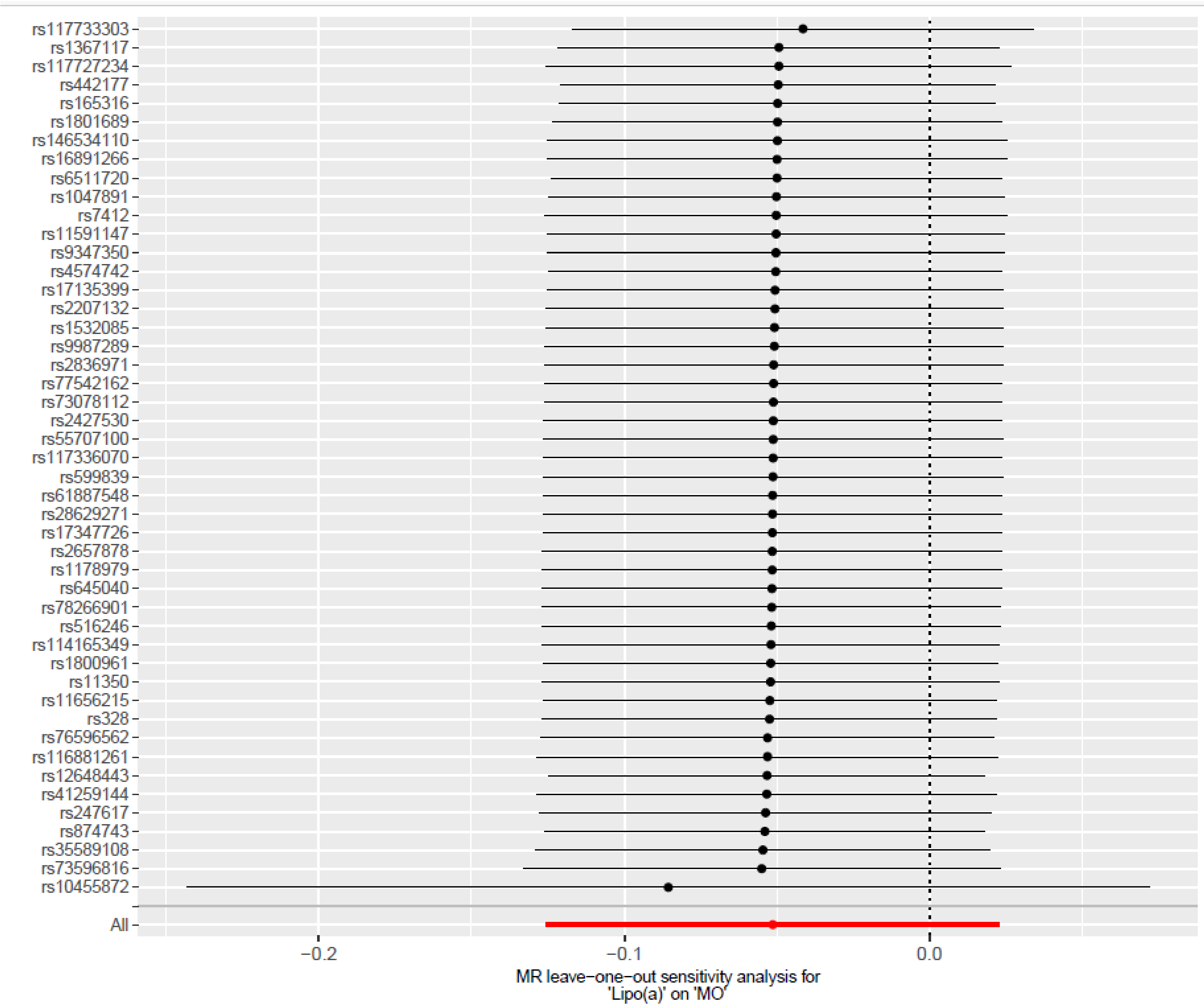

Supplementary Fig. 8. Leave-one-out analysis of MR analyses of (A) LDL-C, (B) HDL-C, (C) TG, (D) TC, (E) ApoB, (F) ApoA1 and (G) lipoprotein (a) on migraine without aura (MO).

(A)

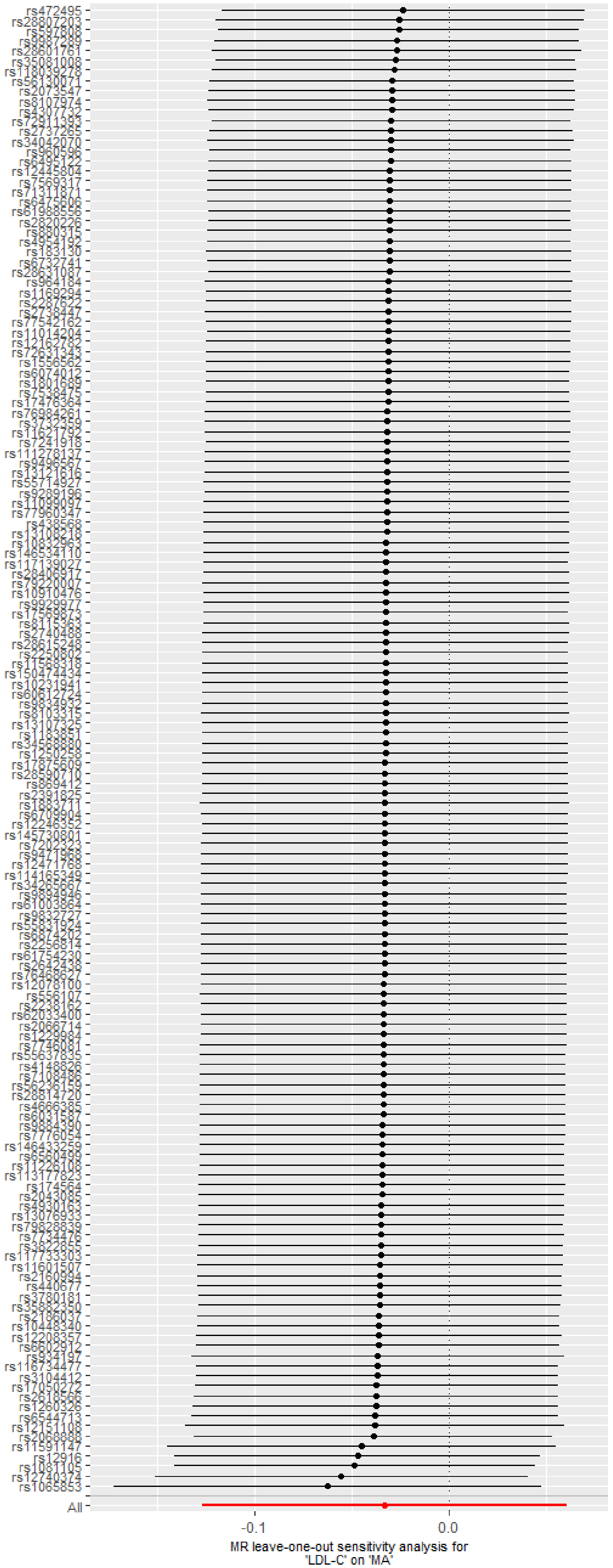
All
-0.1
0.0
MR leave-one-out sensitivity analysis for
'LDL-C' on 'MA'

(B)

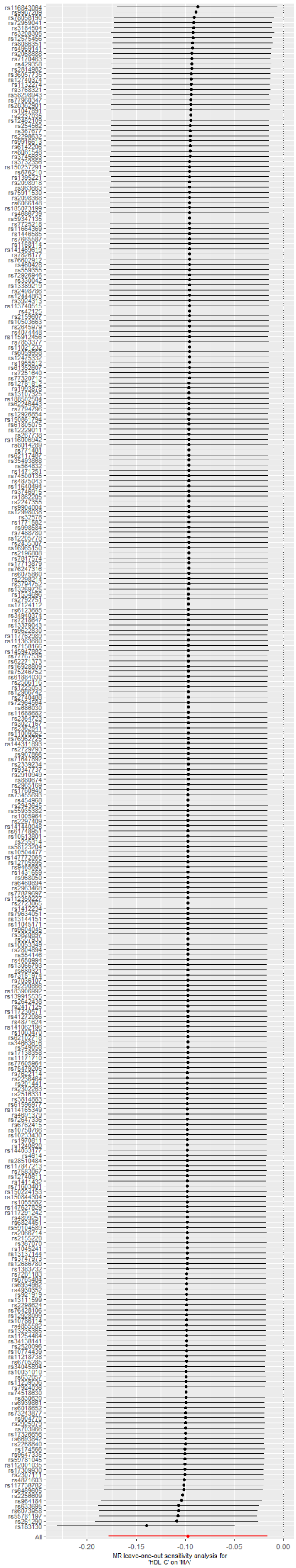
All
-0.20
-0.15
-0.10
-0.05
0.00

(C)

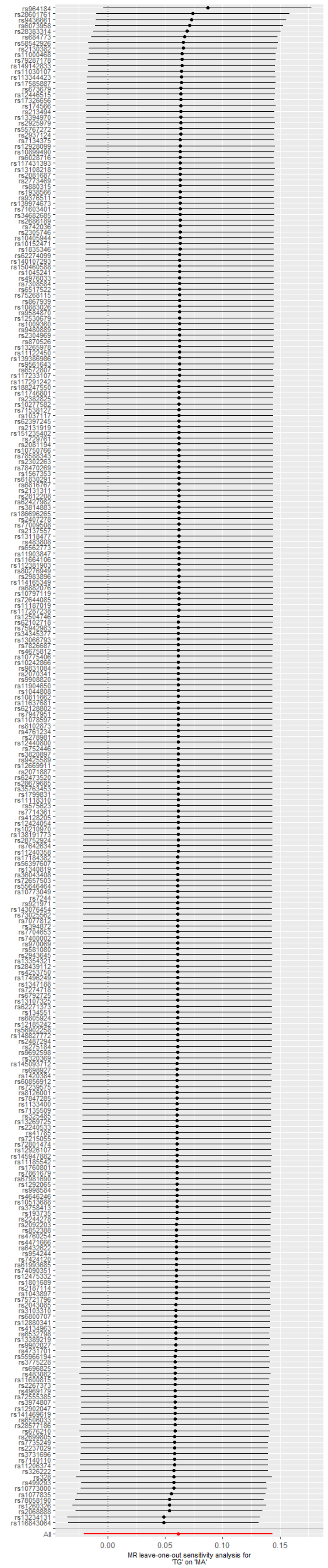

All
0.00
0.05
0.10
0.15
MR leave-one-out sensitivity analysis for
'TG' on 'MA'

(D)

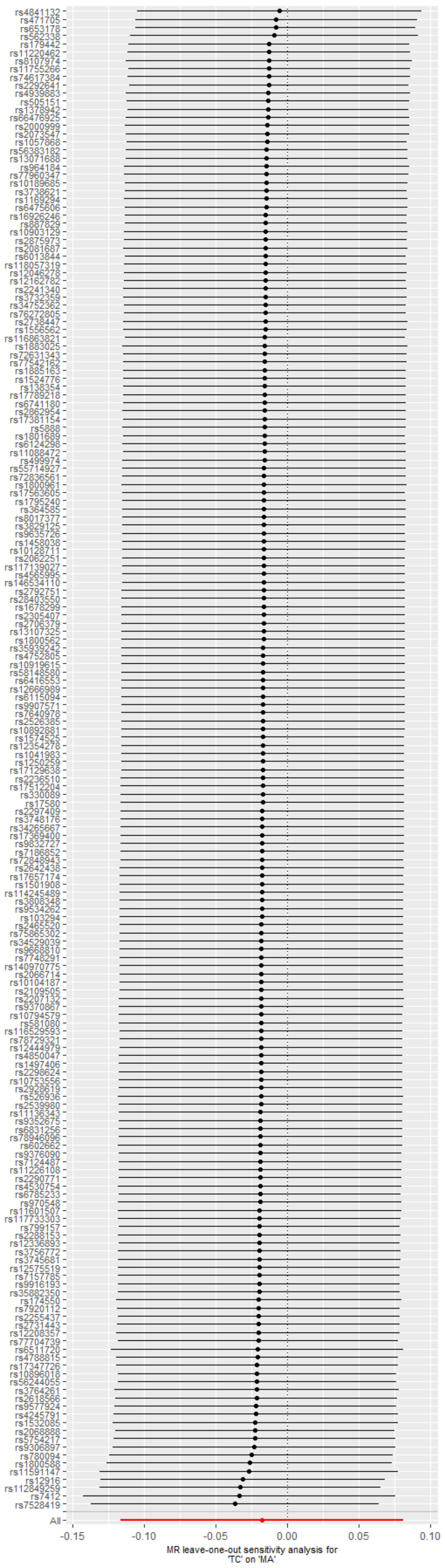

All
-0.15
-0.10
-0.05
0.00
0.05
0.10
MR leave-one-out sensitivity analysis for
'TC' on 'MA'

(E)

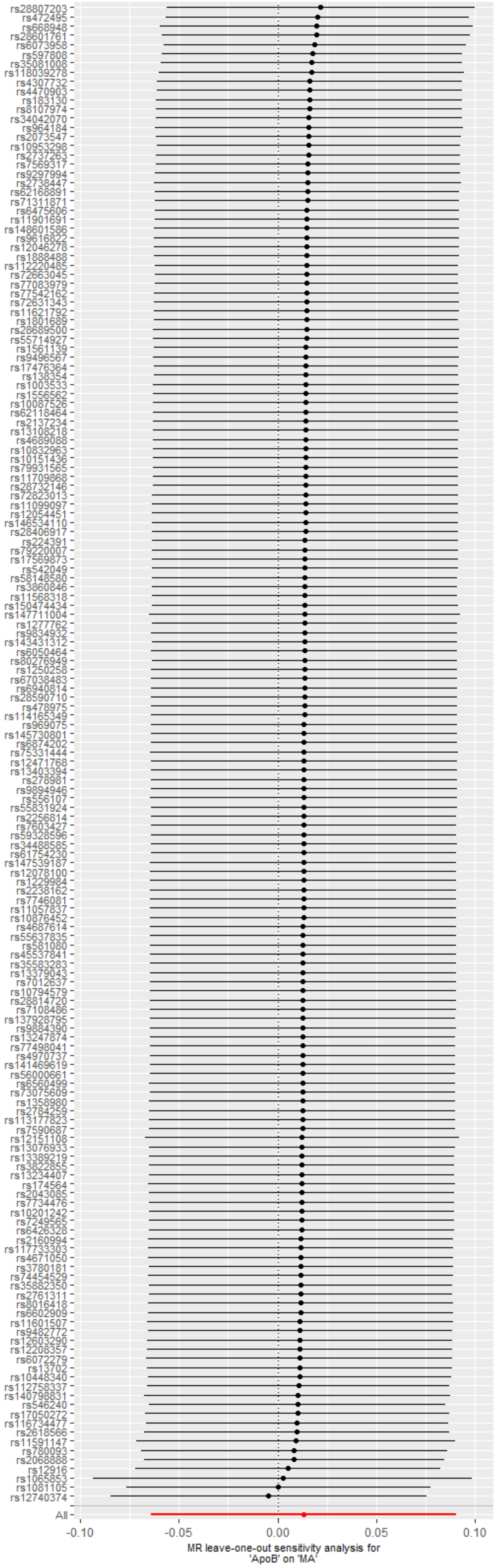
All
-0.10
-0.05
0.00
0.05
0.10
MR leave-one-out sensitivity analysis for
'ApoB' on 'MA'

(F)

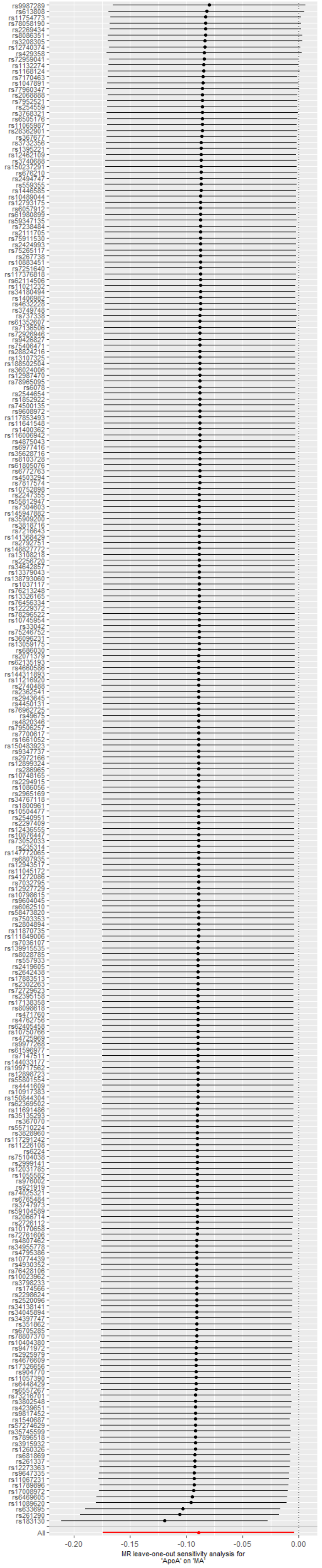
All
-0.20
-0.15
-0.10
-0.05
0.00
MR leave-one-out sensitivity analysis for
'ApoA' on 'MA'

(G)

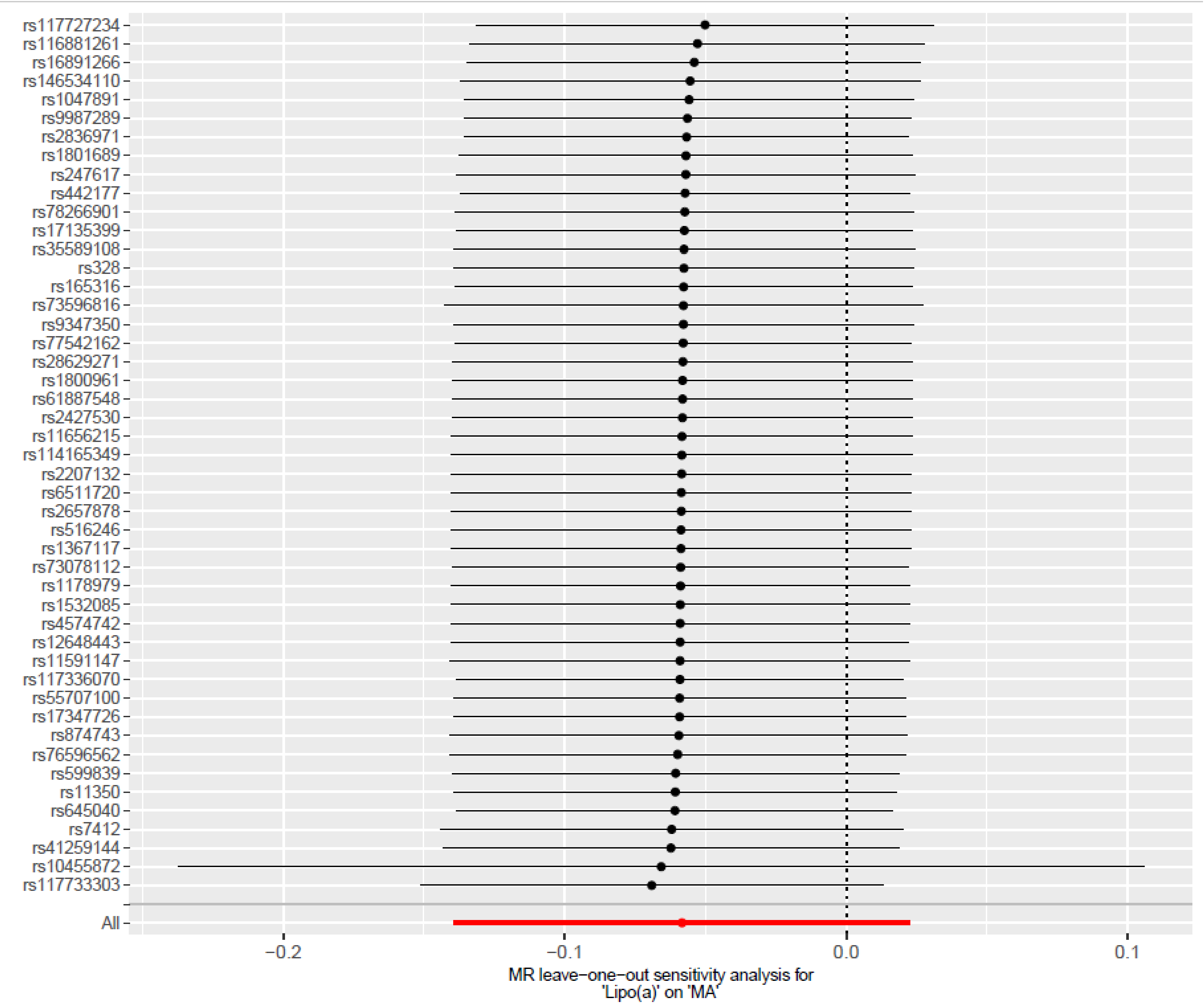

Supplementary Fig. 9. Leave-one-out analysis of MR analyses of (A) LDL-C, (B) HDL-C, (C) TG, (D) TC, (E) ApoB, (F) ApoA1 and (G) lipoprotein (a) on migraine with aura (MA).

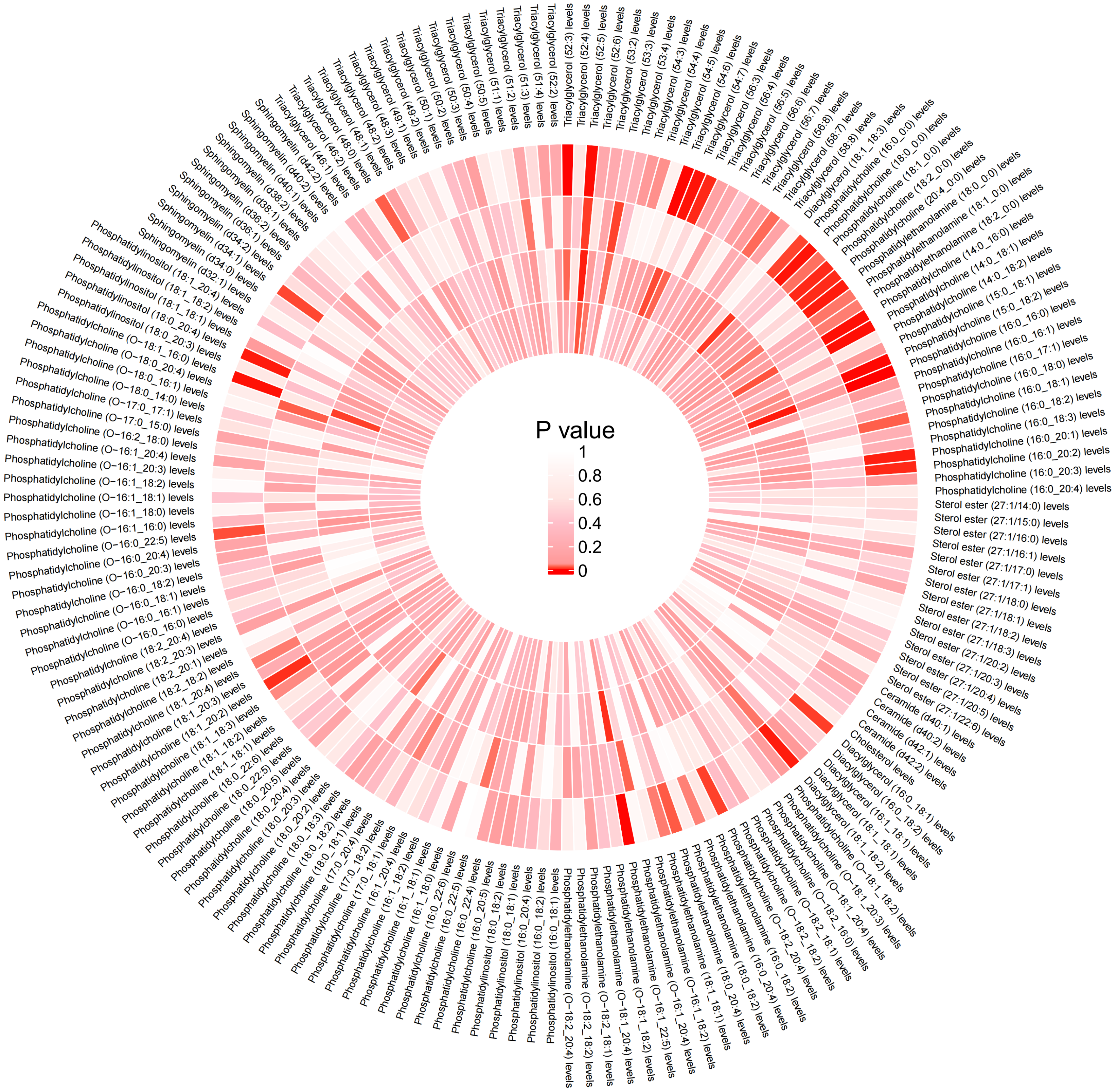

Supplementary Fig. 10. Circular heatmap of the causal effect of 179 lipid species components on overall migraine. The circular heat map represents the four MR methods, including IVW, Weighted Median, MR-Egger, Weighted mode, in order from the outer ring to the inner ring.

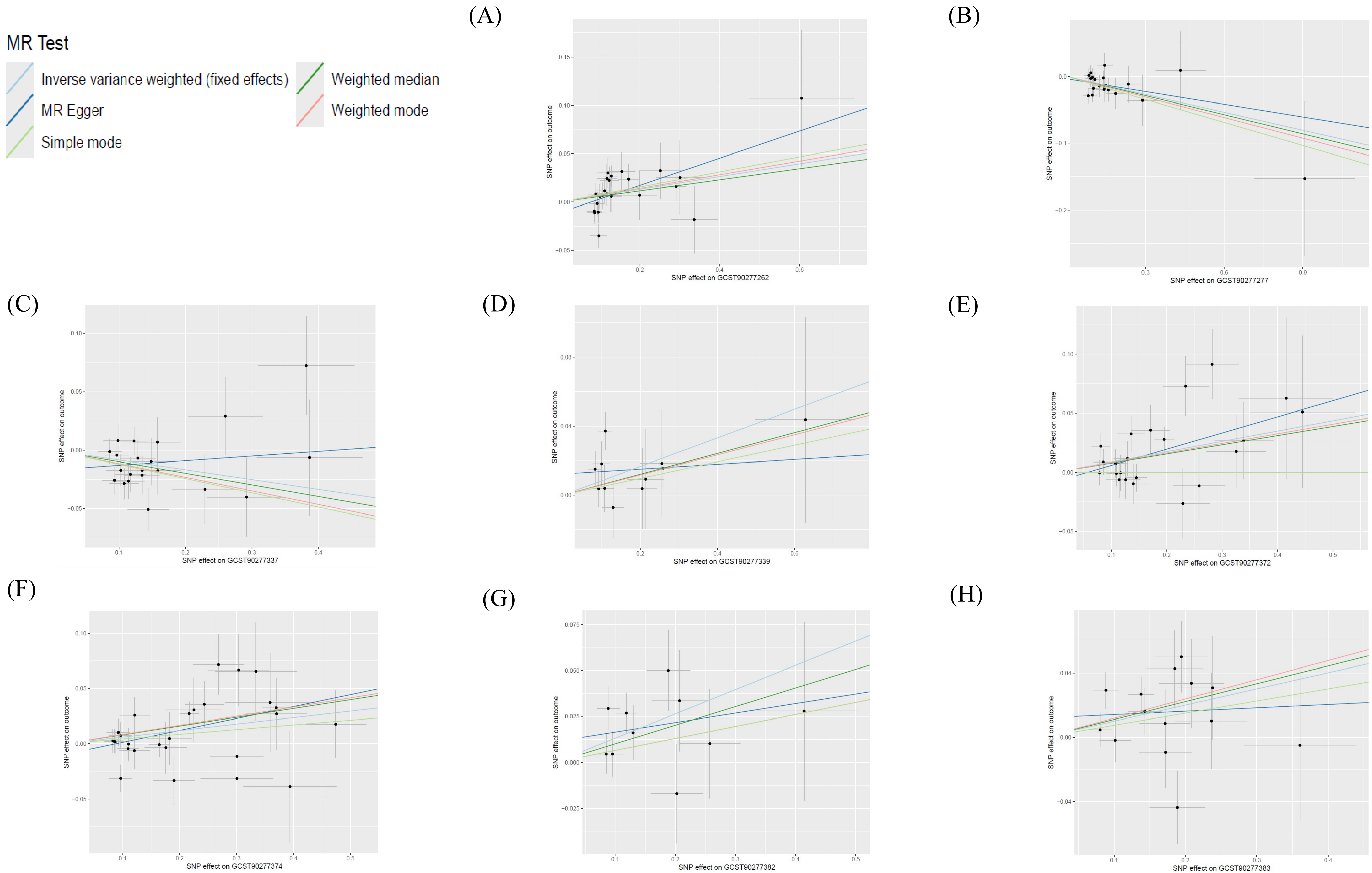

MR Test
Inverse variance weighted (fixed effects)
MR Egger
Simple mode
Weighted median
Weighted mode
(A)
SNP effect on outcome
SNP effect on GCST90277262
(B)
SNP effect on outcome
SNP effect on GCST90277277
(C)
SNP effect on outcome
SNP effect on GCST90277337
(D)
SNP effect on outcome
SNP effect on GCST90277339
(E)
SNP effect on outcome
SNP effect on GCST90277372
(F)
SNP effect on outcome
SNP effect on GCST90277374
(G)
SNP effect on outcome
SNP effect on GCST90277382
(H)
SNP effect on outcome
SNP effect on GCST90277383

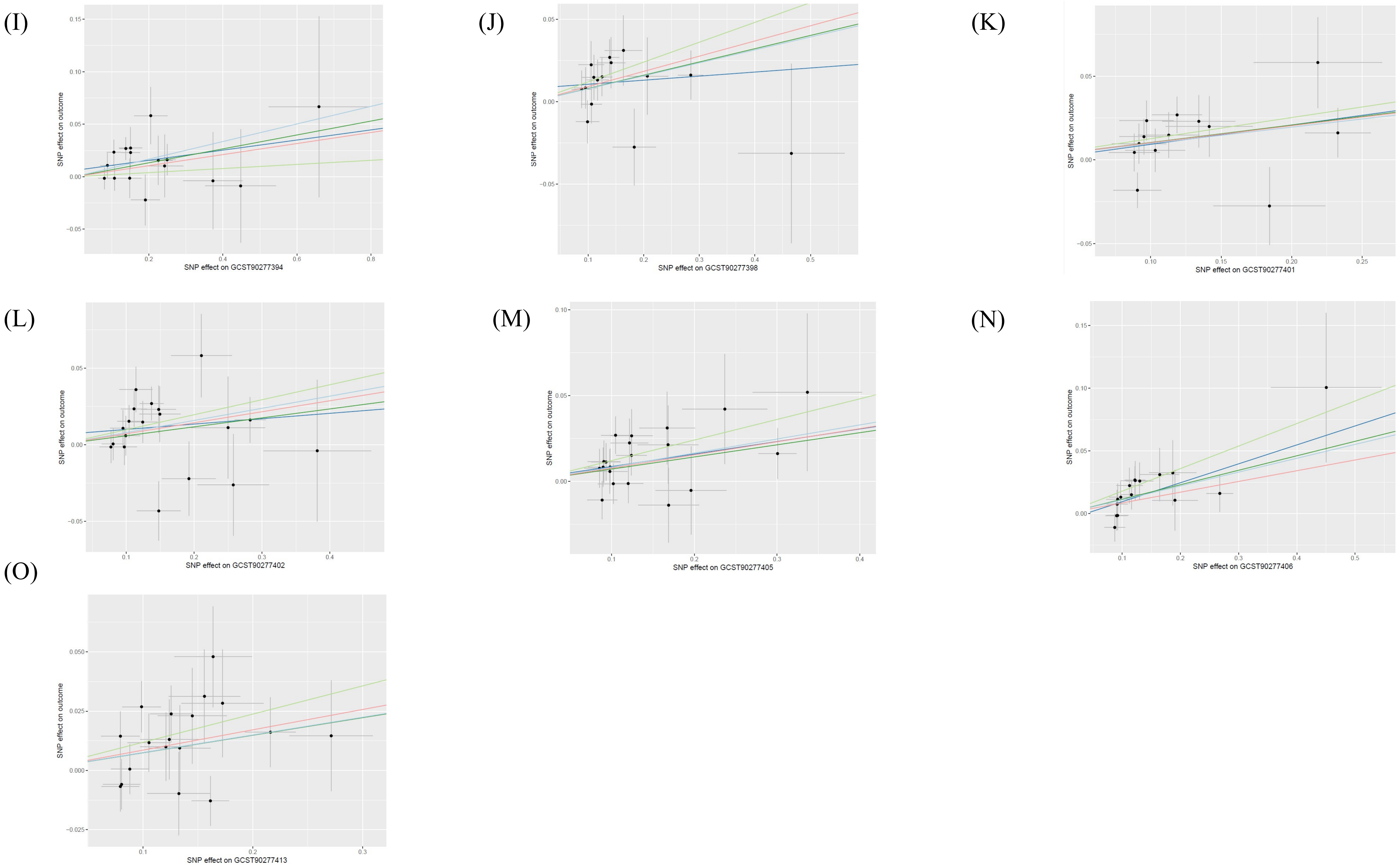

Supplementary Fig. 11. Scatter plots to show MR estimates of (A) DAG(18:1_18:2), (B) PEO (O-16:1_22:5), (C) PC(O-18:1_16:0), (D) PI(18:0_20:4), (E) TAG(52:3), (F) TAG(52:5), (G) TAG(54:6), (H) TAG(54:7), (I) PC(18:0_0:0),(J) PE(18:0_0:0), (K) PC(14:0_16:0), (L) PC(14:0_18:1), (M) PC(15:0_18:2), (N) PC(16:0_16:0) and (O) PC(16:0_20:1) on migraine.

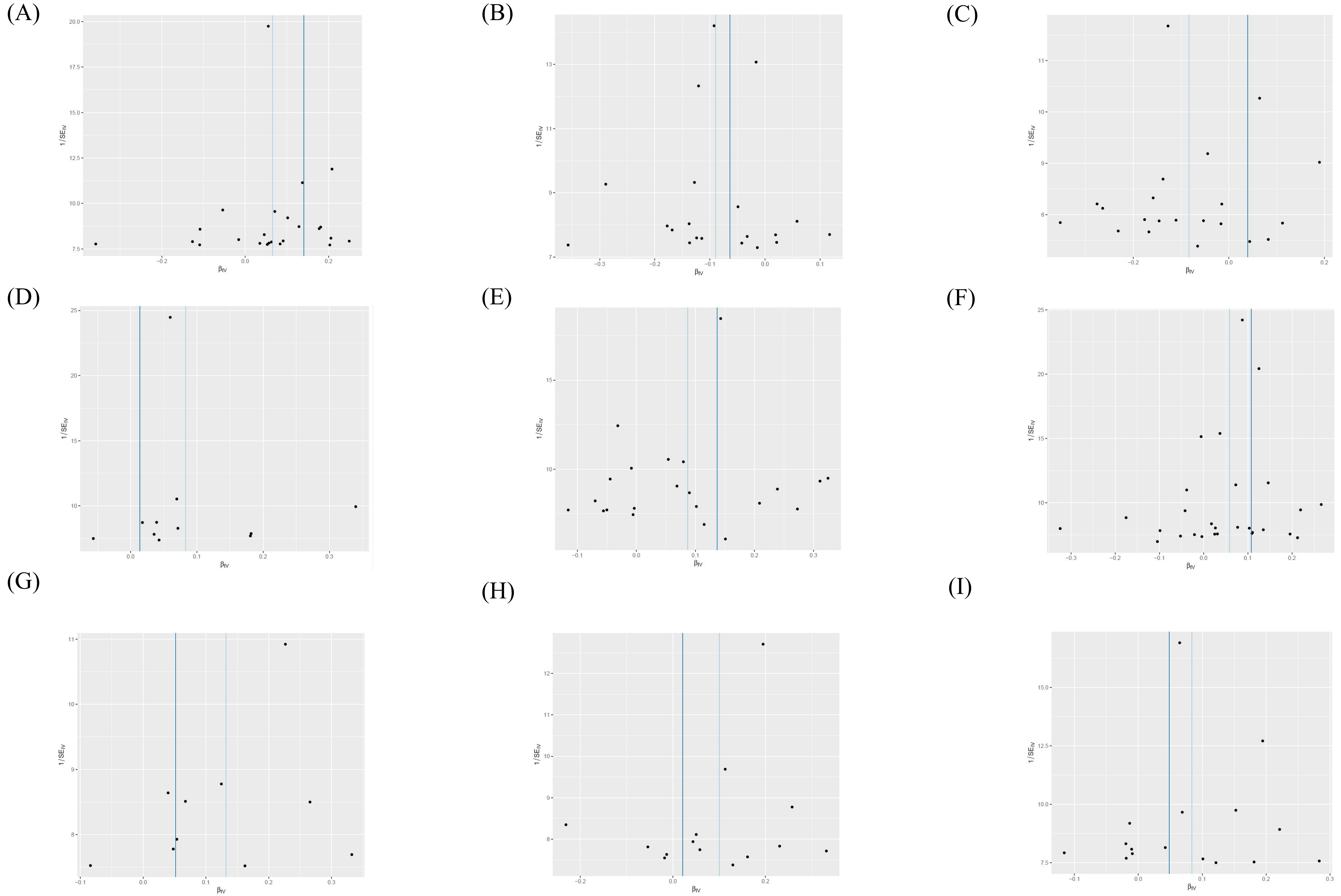
(A)
(B)
(C)
(D)
(E)
(F)
(G)
(H)
(I)
1/SE_IV
β_IV

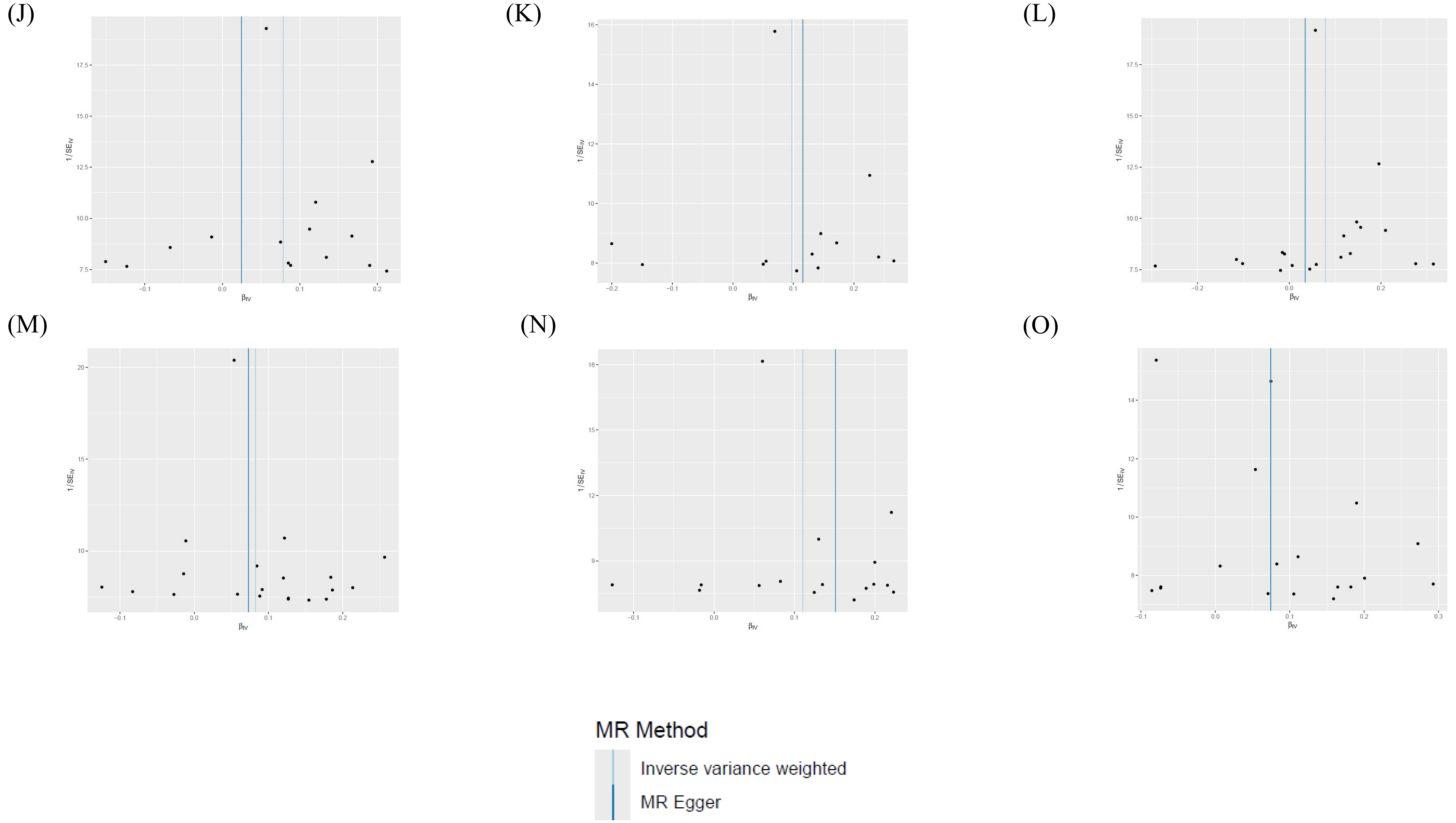

Supplementary Fig. 12. Funnel plots of MR analyses of (A) DAG(18:1_18:2), (B)PEO (O-16:1_22:5), (C) PC(O-18:1_16:0), (D) PI(18:0_20:4), (E) TAG(52:3), (F) TAG(52:5), (G) TAG(54:6), (H) TAG(54:7), (I) PC(18:0_0:0),(J) PE(18:0_0:0), (K) PC(14:0_16:0), (L) PC(14:0_18:1), (M) PC(15:0_18:2), (N) PC(16:0_16:0) and (O) PC(16:0_20:1) on migraine.

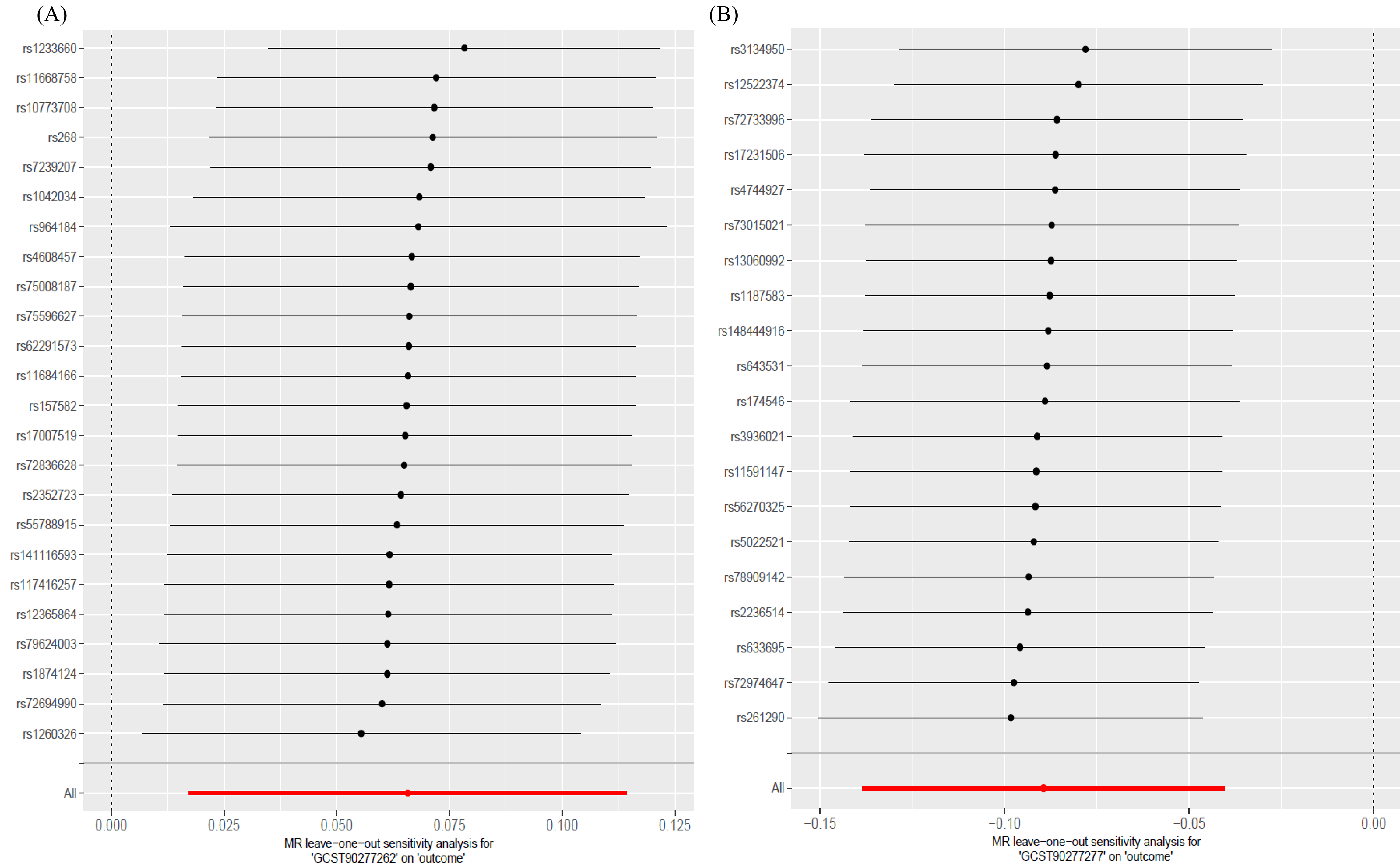
(A)
rs1233660
rs11668758
rs10773708
rs268
rs7239207
rs1042034
rs964184
rs4608457
rs75008187
rs75596627
rs62291573
rs11684166
rs157582
rs17007519
rs72836628
rs2352723
rs55788915
rs141116593
rs117416257
rs12365864
rs79624003
rs1874124
rs72694990
rs1260326
All
0.000
0.025
0.050
0.075
0.100
0.125
MR leave−one−out sensitivity analysis for
'GCST90277262' on 'outcome'
(B)
rs3134950
rs12522374
rs72733996
rs17231506
rs4744927
rs73015021
rs13060992
rs1187583
rs148444916
rs643531
rs174546
rs3936021
rs11591147
rs56270325
rs5022521
rs78909142
rs2236514
rs633695
rs72974647
rs261290
All
−0.15
−0.10
−0.05
0.00
MR leave−one−out sensitivity analysis for
'GCST90277277' on 'outcome'

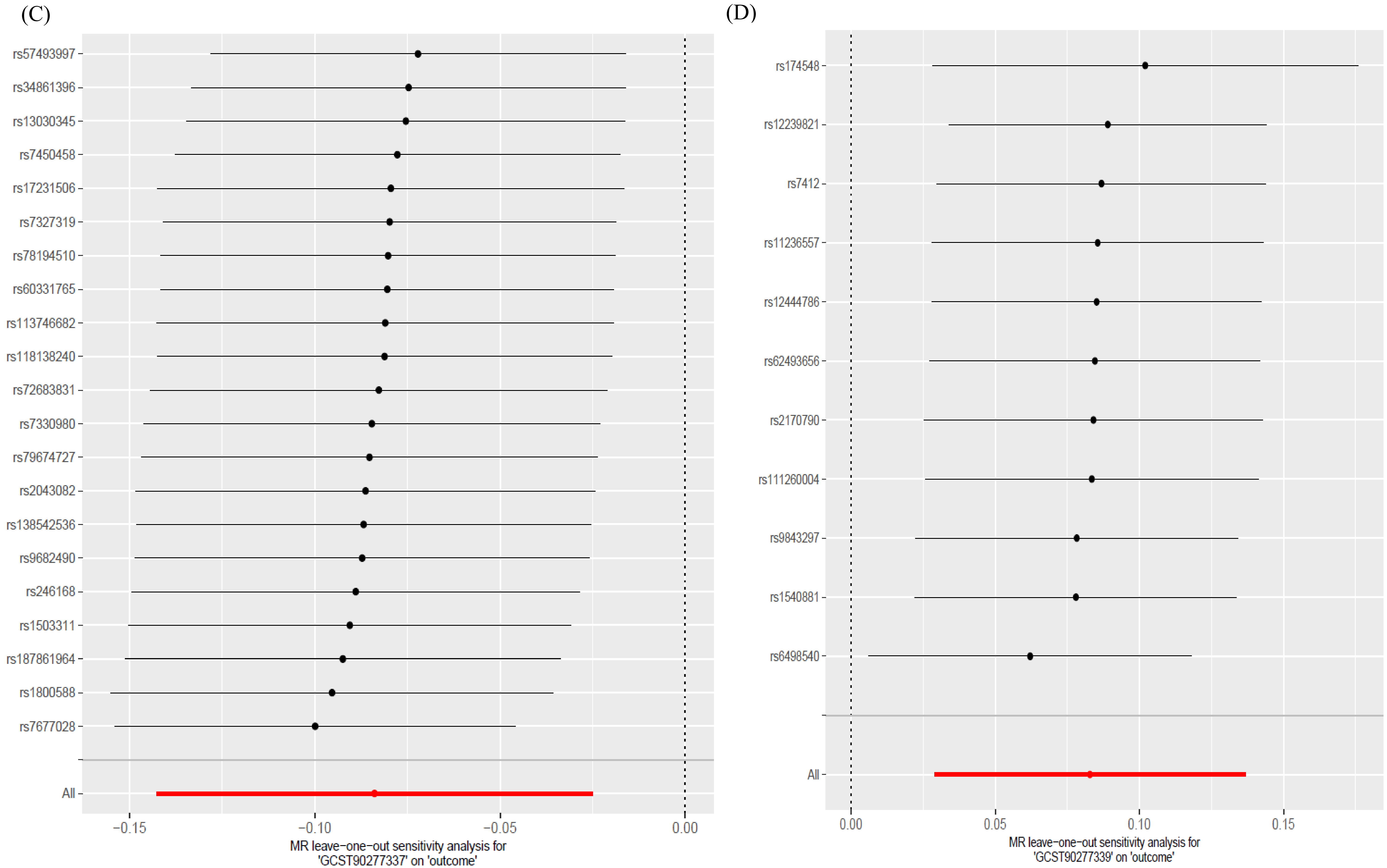

(C)
rs57493997
rs34861396
rs13030345
rs7450458
rs17231506
rs7327319
rs78194510
rs60331765
rs113746682
rs118138240
rs72683831
rs7330980
rs79674727
rs2043082
rs138542536
rs9682490
rs246168
rs1503311
rs187861964
rs1800588
rs7677028
All
−0.15
−0.10
−0.05
0.00
MR leave−one−out sensitivity analysis for
'GCST90277337' on 'outcome'
(D)
rs174548
rs12239821
rs7412
rs11236557
rs12444786
rs62493656
rs2170790
rs111260004
rs9843297
rs1540881
rs6498540
All
0.00
0.05
0.10
0.15
MR leave−one−out sensitivity analysis for
'GCST90277339' on 'outcome'

(E)

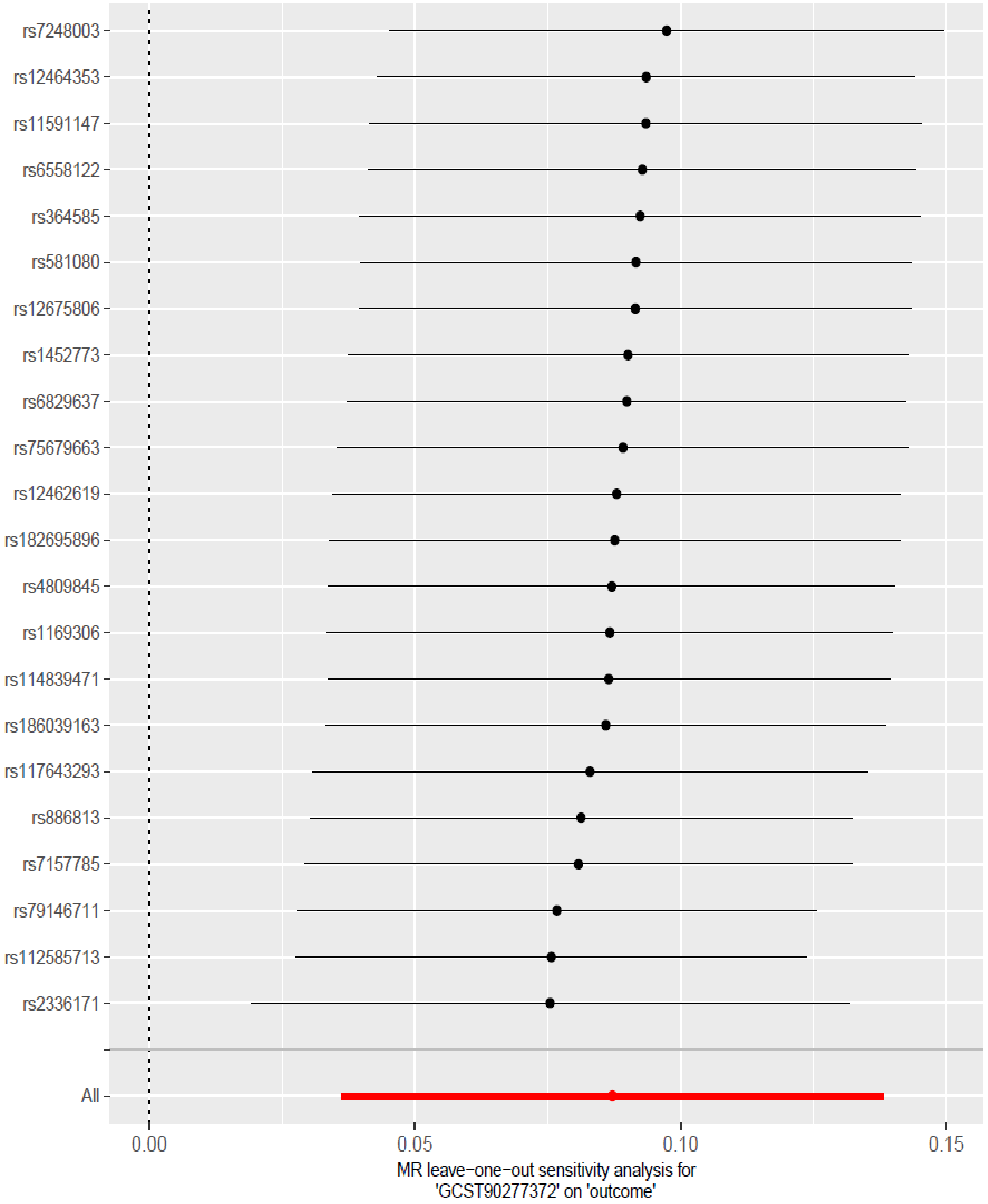
rs7248003
rs12464353
rs11591147
rs6558122
rs364585
rs581080
rs12675806
rs1452773
rs6829637
rs75679663
rs12462619
rs182695896
rs4809845
rs1169306
rs114839471
rs186039163
rs117643293
rs886813
rs7157785
rs79146711
rs112585713
rs2336171
All
0.00
0.05
0.10
0.15
MR leave−one−out sensitivity analysis for
'GCST90277372' on 'outcome'

(F)

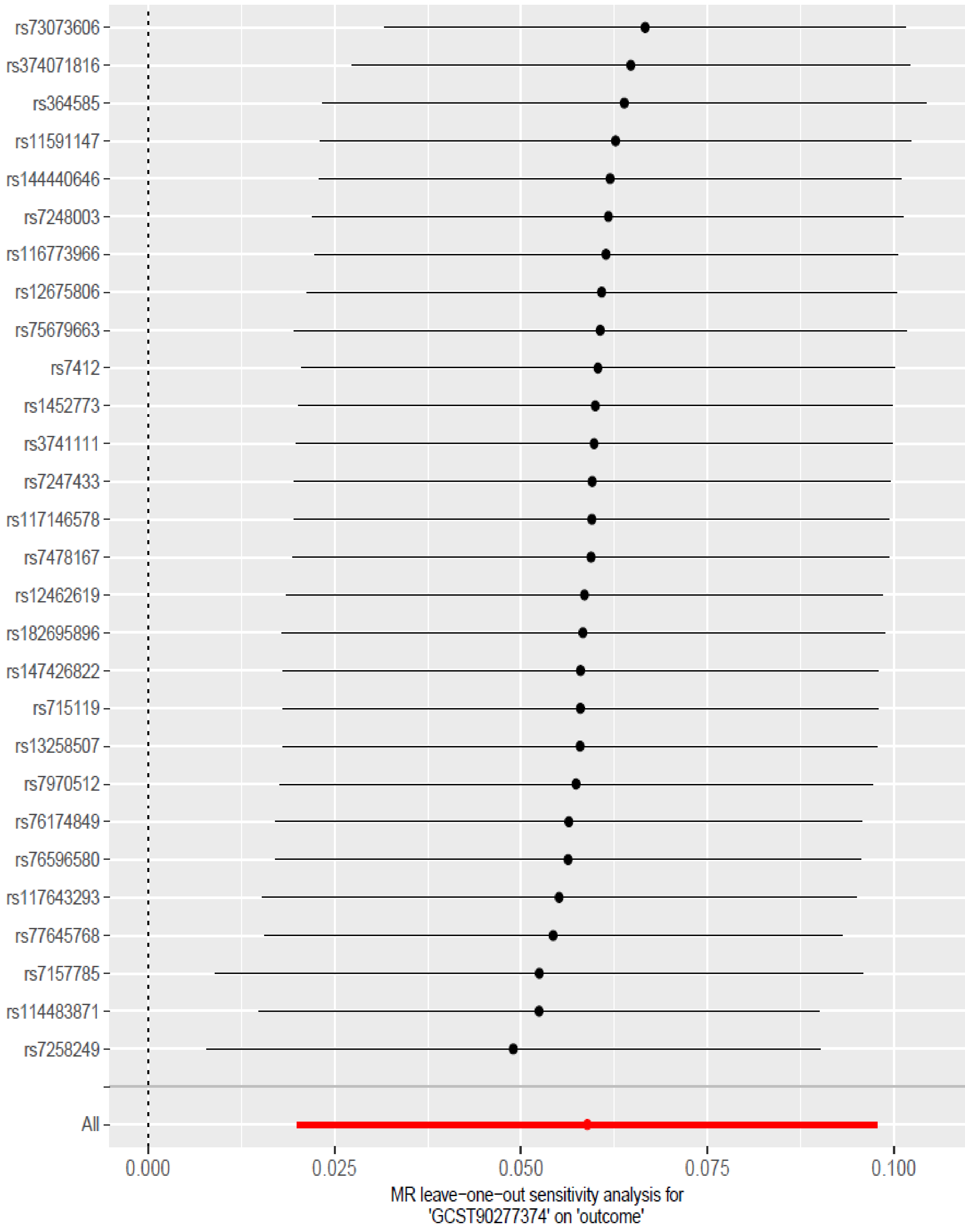
rs73073606
rs374071816
rs364585
rs11591147
rs144440646
rs7248003
rs116773966
rs12675806
rs75679663
rs7412
rs1452773
rs3741111
rs7247433
rs117146578
rs7478167
rs12462619
rs182695896
rs147426822
rs715119
rs13258507
rs7970512
rs76174849
rs76596580
rs117643293
rs77645768
rs7157785
rs114483871
rs7258249
All
0.000
0.025
0.050
0.075
0.100
MR leave−one−out sensitivity analysis for
'GCST90277374' on 'outcome'

(G)

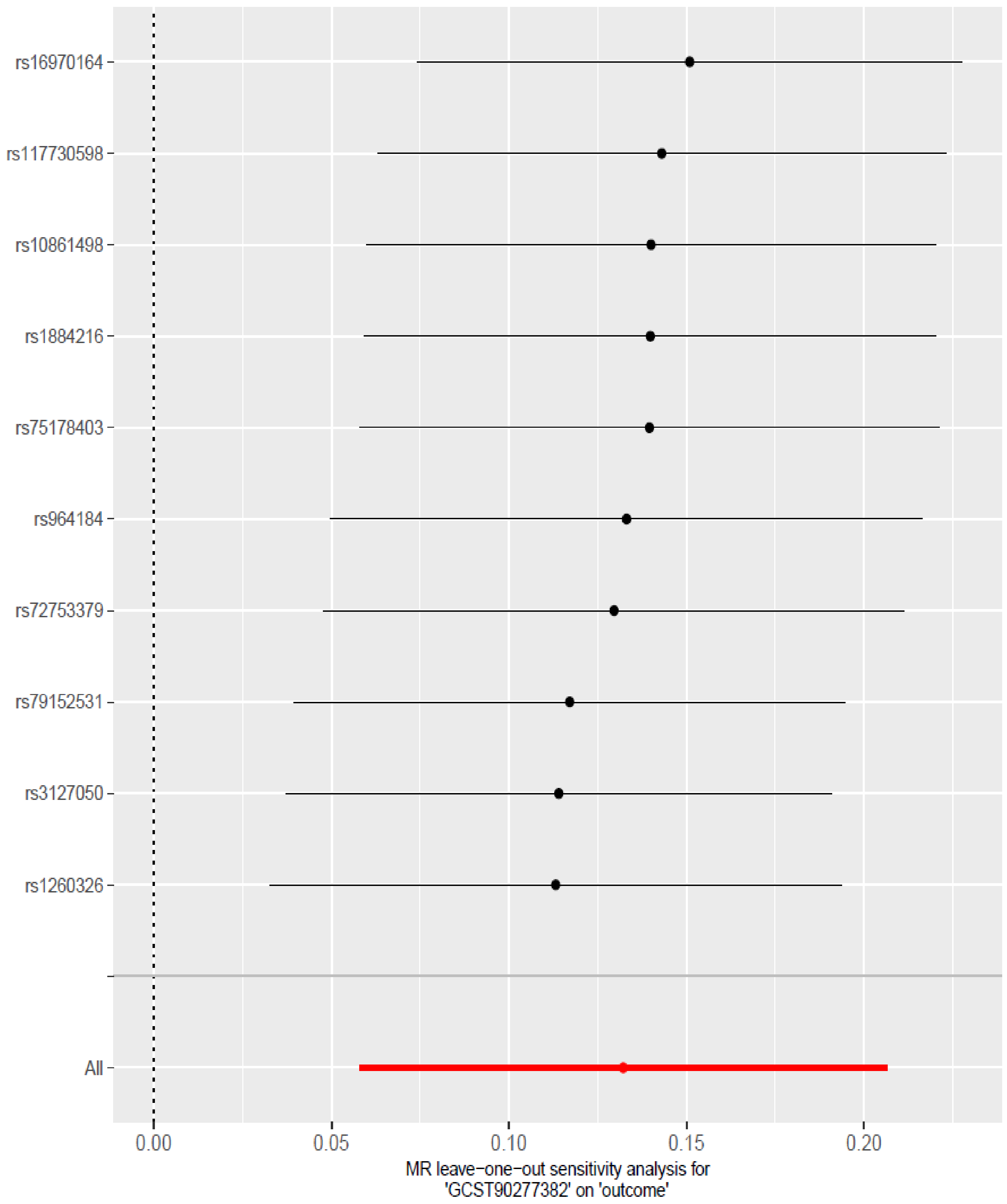
rs16970164
rs117730598
rs10861498
rs1884216
rs75178403
rs964184
rs72753379
rs79152531
rs3127050
rs1260326
All
0.00
0.05
0.10
0.15
0.20
MR leave−one−out sensitivity analysis for
'GCST90277382' on 'outcome'

(H)

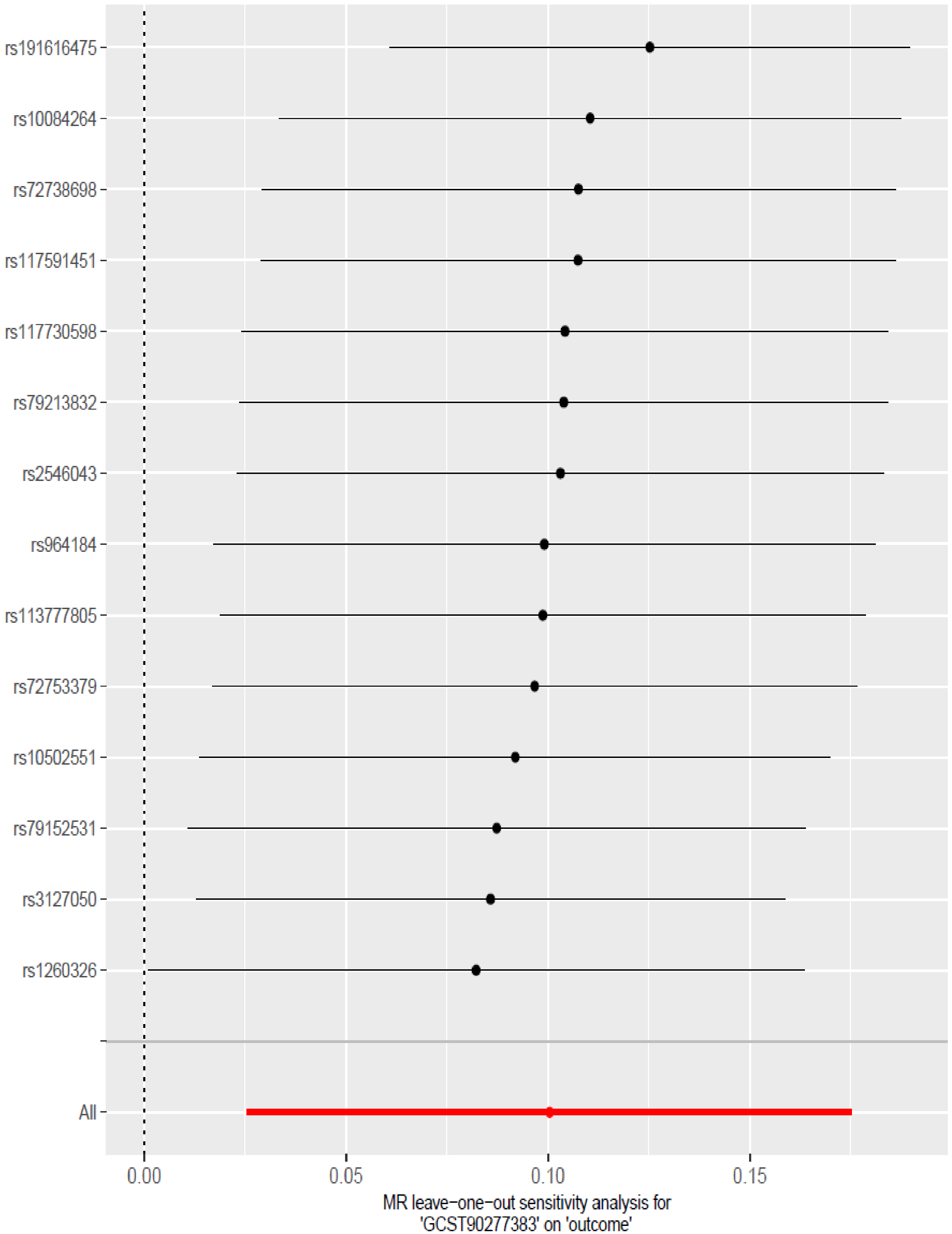
rs191616475
rs10084264
rs72738698
rs117591451
rs117730598
rs79213832
rs2546043
rs964184
rs113777805
rs72753379
rs10502551
rs79152531
rs3127050
rs1260326
All
0.00
0.05
0.10
0.15
MR leave−one−out sensitivity analysis for
'GCST90277383' on 'outcome'

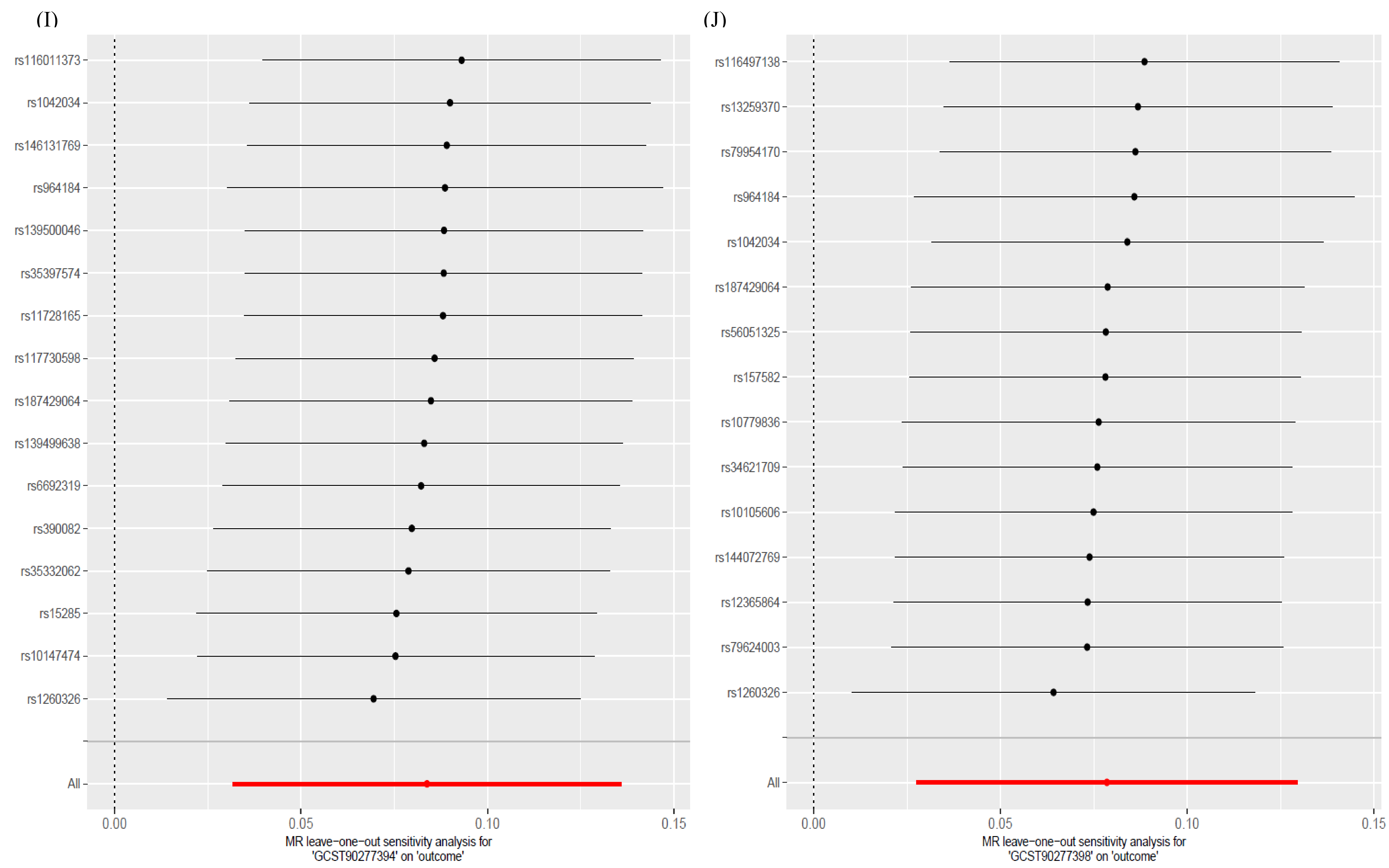
(I)
rs116011373
rs1042034
rs146131769
rs964184
rs139500046
rs35397574
rs11728165
rs117730598
rs187429064
rs139499638
rs6692319
rs390082
rs35332062
rs15285
rs10147474
rs1260326
All
0.00
0.05
0.10
0.15
MR leave−one−out sensitivity analysis for
'GCST90277394' on 'outcome'
(J)
rs116497138
rs13259370
rs79954170
rs964184
rs1042034
rs187429064
rs56051325
rs157582
rs10779836
rs34621709
rs10105606
rs144072769
rs12365864
rs79624003
rs1260326
All
0.00
0.05
0.10
0.15
MR leave−one−out sensitivity analysis for
'GCST90277398' on 'outcome'

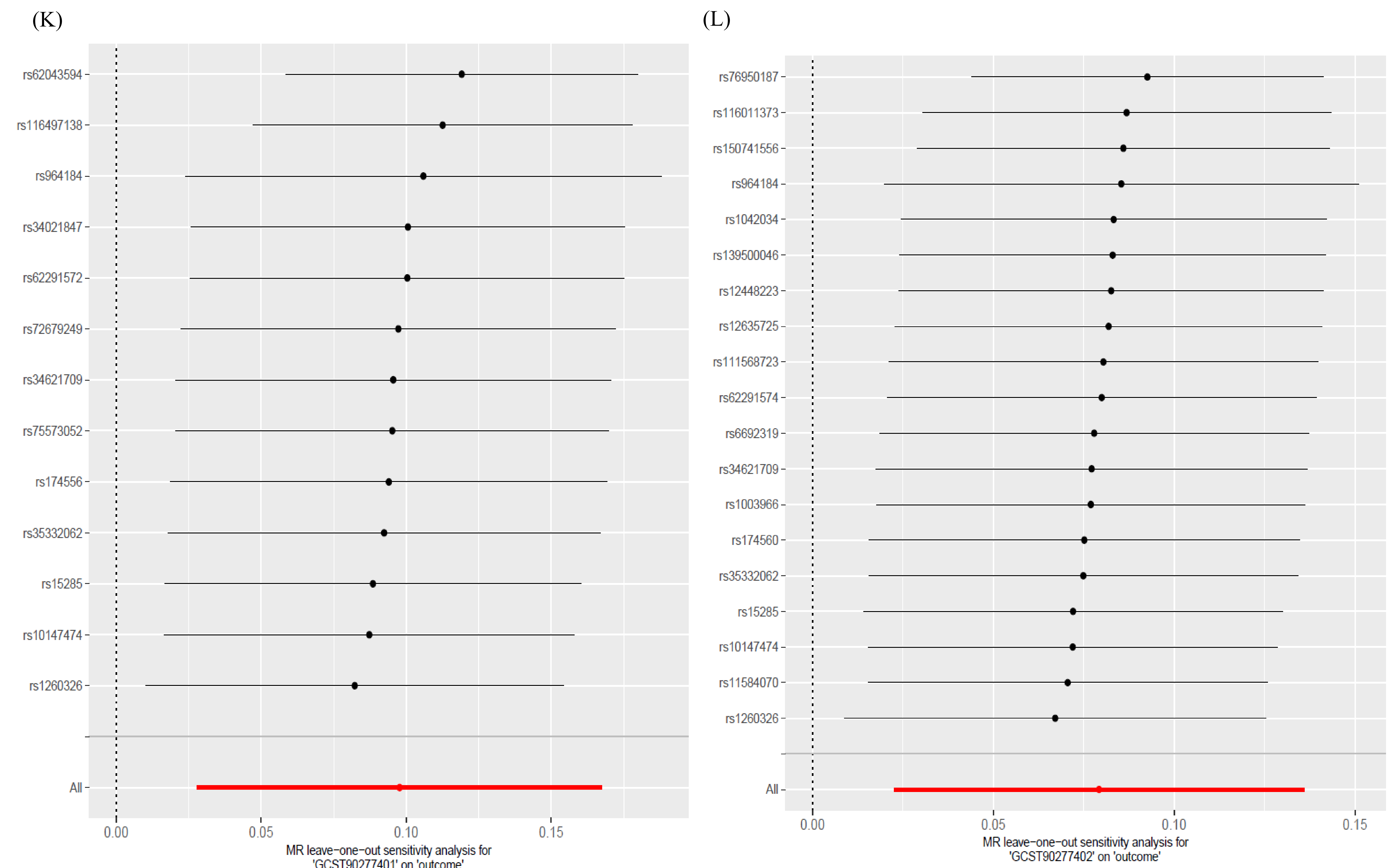
(K)
rs62043594
rs116497138
rs964184
rs34021847
rs62291572
rs72679249
rs34621709
rs75573052
rs174556
rs35332062
rs15285
rs10147474
rs1260326
All
0.00
0.05
0.10
0.15
MR leave−one−out sensitivity analysis for
'GCST90277401' on 'outcome'
(L)
rs76950187
rs116011373
rs150741556
rs964184
rs1042034
rs139500046
rs12448223
rs12635725
rs111568723
rs62291574
rs6692319
rs34621709
rs1003966
rs174560
rs35332062
rs15285
rs10147474
rs11584070
rs1260326
All
0.00
0.05
0.10
0.15
MR leave−one−out sensitivity analysis for
'GCST90277402' on 'outcome'

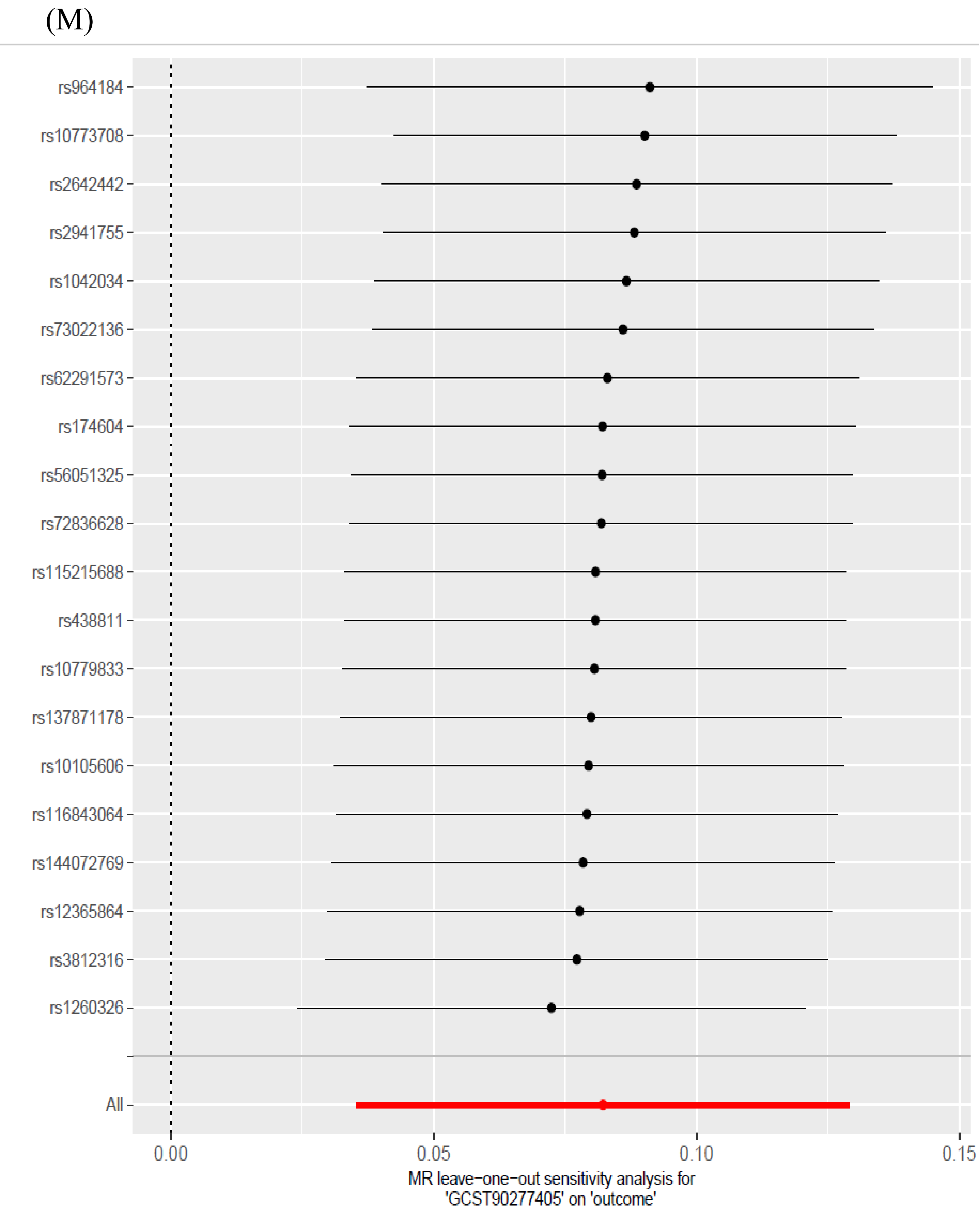
(M)
rs964184
rs10773708
rs2642442
rs2941755
rs1042034
rs73022136
rs62291573
rs174604
rs56051325
rs72836628
rs115215688
rs438811
rs10779833
rs137871178
rs10105606
rs116843064
rs144072769
rs12365864
rs3812316
rs1260326
All
0.00
0.05
0.10
0.15
MR leave−one−out sensitivity analysis for
'GCST90277405' on 'outcome'

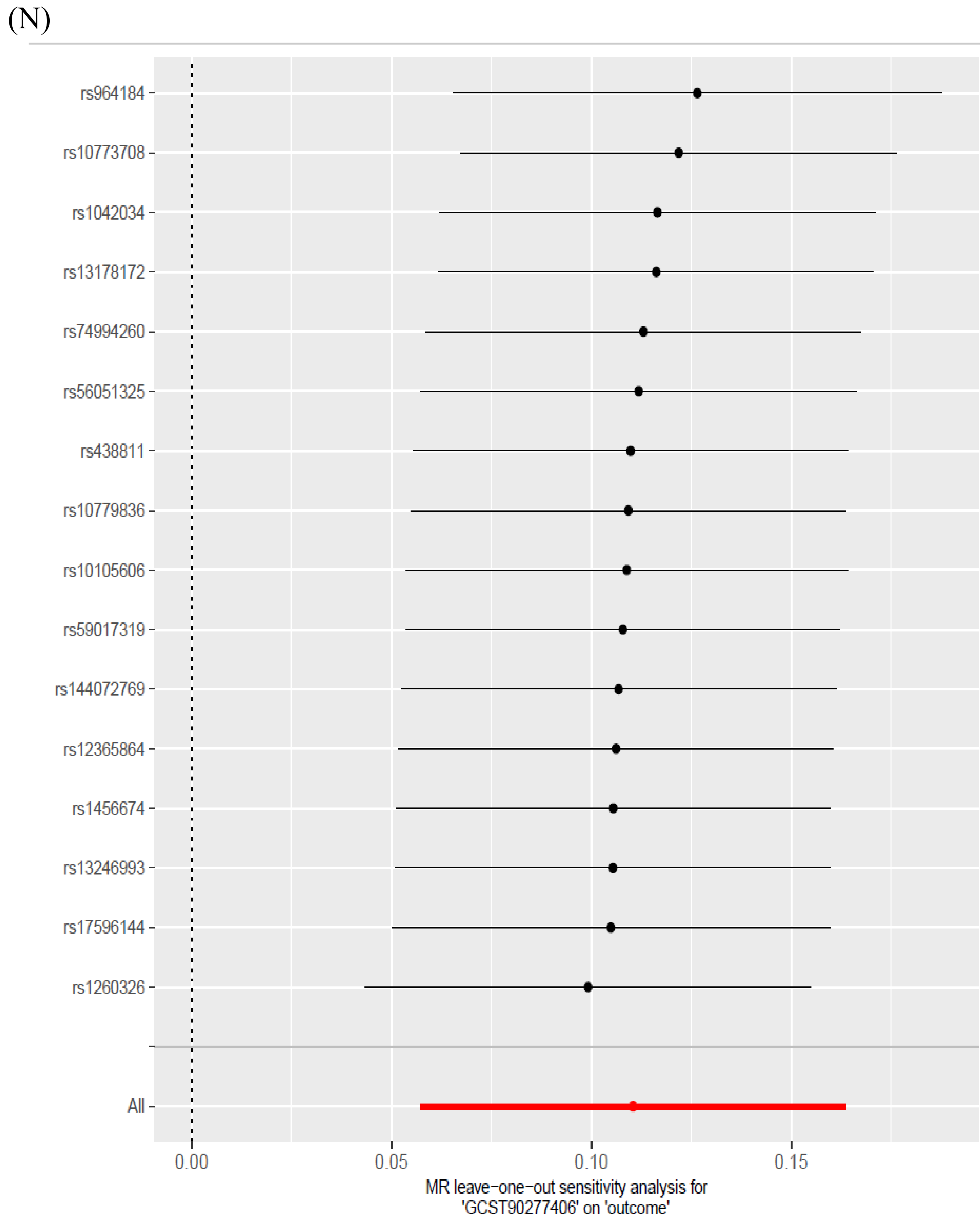
(N)
rs964184
rs10773708
rs1042034
rs13178172
rs74994260
rs56051325
rs438811
rs10779836
rs10105606
rs59017319
rs144072769
rs12365864
rs1456674
rs13246993
rs17596144
rs1260326
All
0.00
0.05
0.10
0.15
MR leave−one−out sensitivity analysis for
'GCST90277406' on 'outcome'

(O)

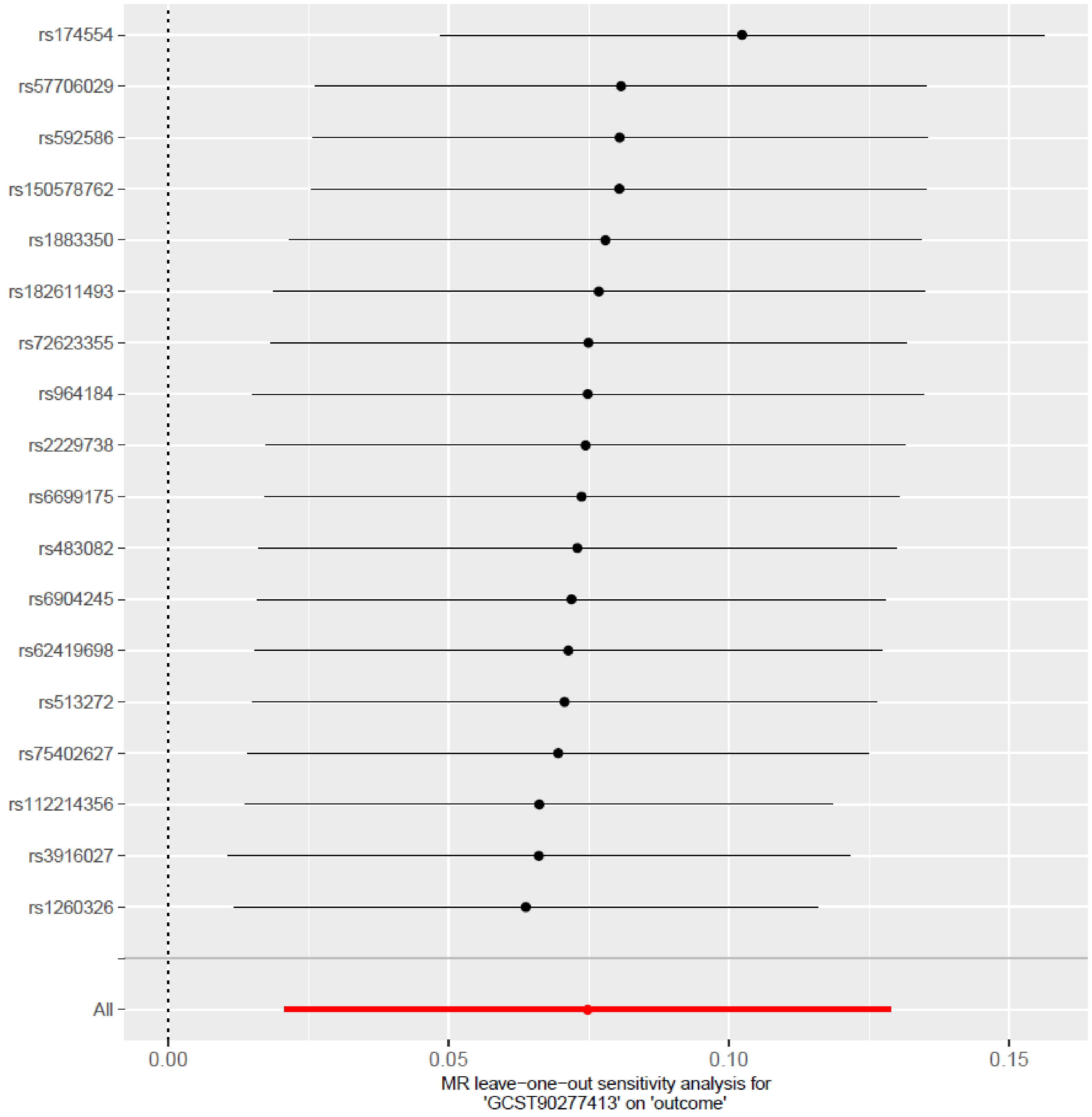

Supplementary Fig. 13. Leave-one-out analysis of MR analyses of (A) DAG(18:1_18:2), (B)PEO (O-16:1_22:5), (C) PC(O-18:1_16:0), (D) PI(18:0_20:4), (E) TAG(52:3), (F) TAG(52:5), (G) TAG(54:6), (H) TAG(54:7), (I) PC(18:0_0:0),(J) PE(18:0_0:0), (K) PC(14:0_16:0), (L) PC(14:0_18:1), (M)
PC(15:0_18:2), (N) PC(16:0_16:0) and (O) PC(16:0_20:1) on migraine.

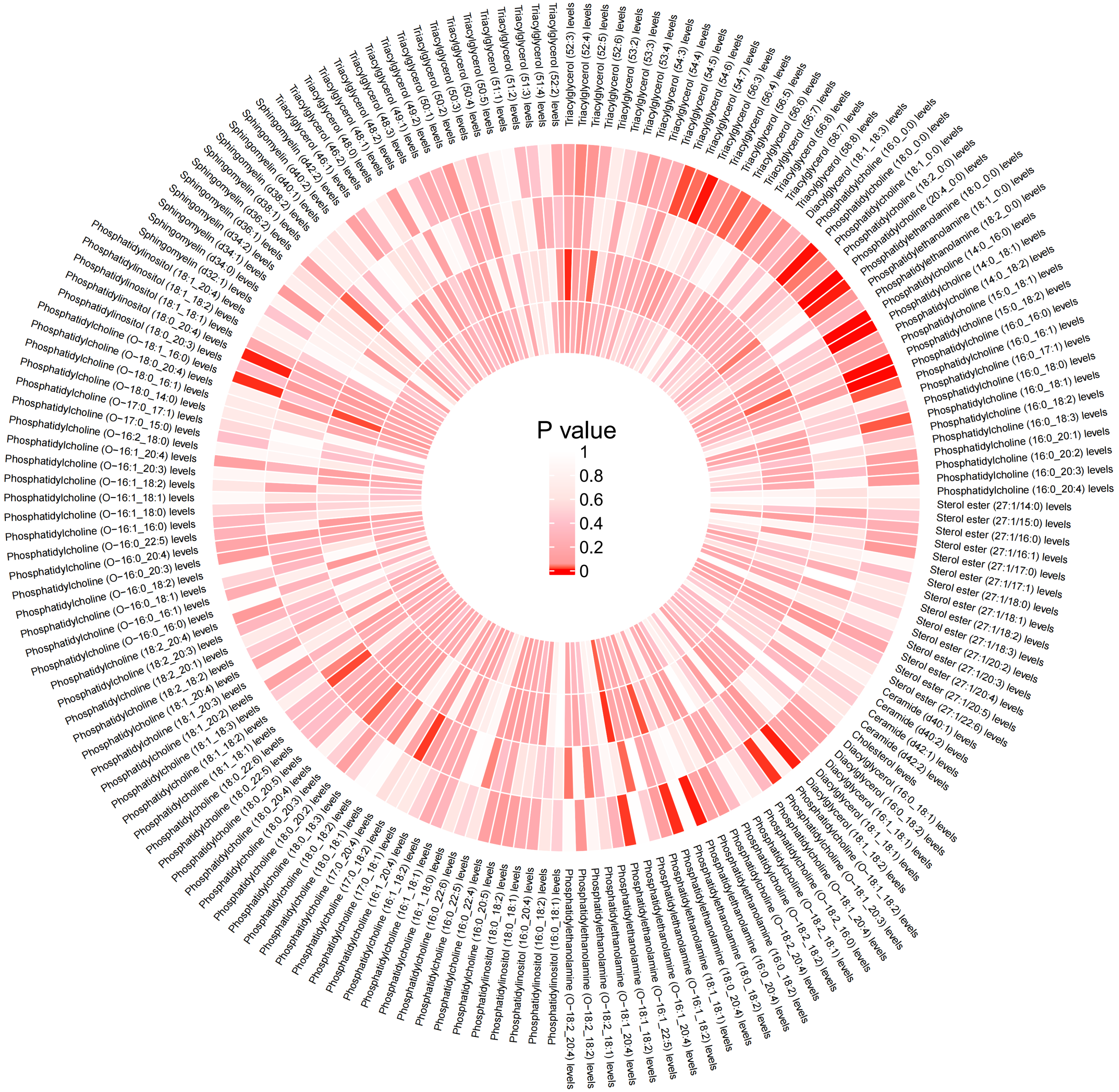

Supplementary Fig. 14. Circular heatmap of the causal effect of 179 lipid species components on migraine without aura. The circular heat map represents the four MR methods, including IVW, Weighted Median, MR-Egger, Weighted mode, in order from the outer ring to the inner ring.

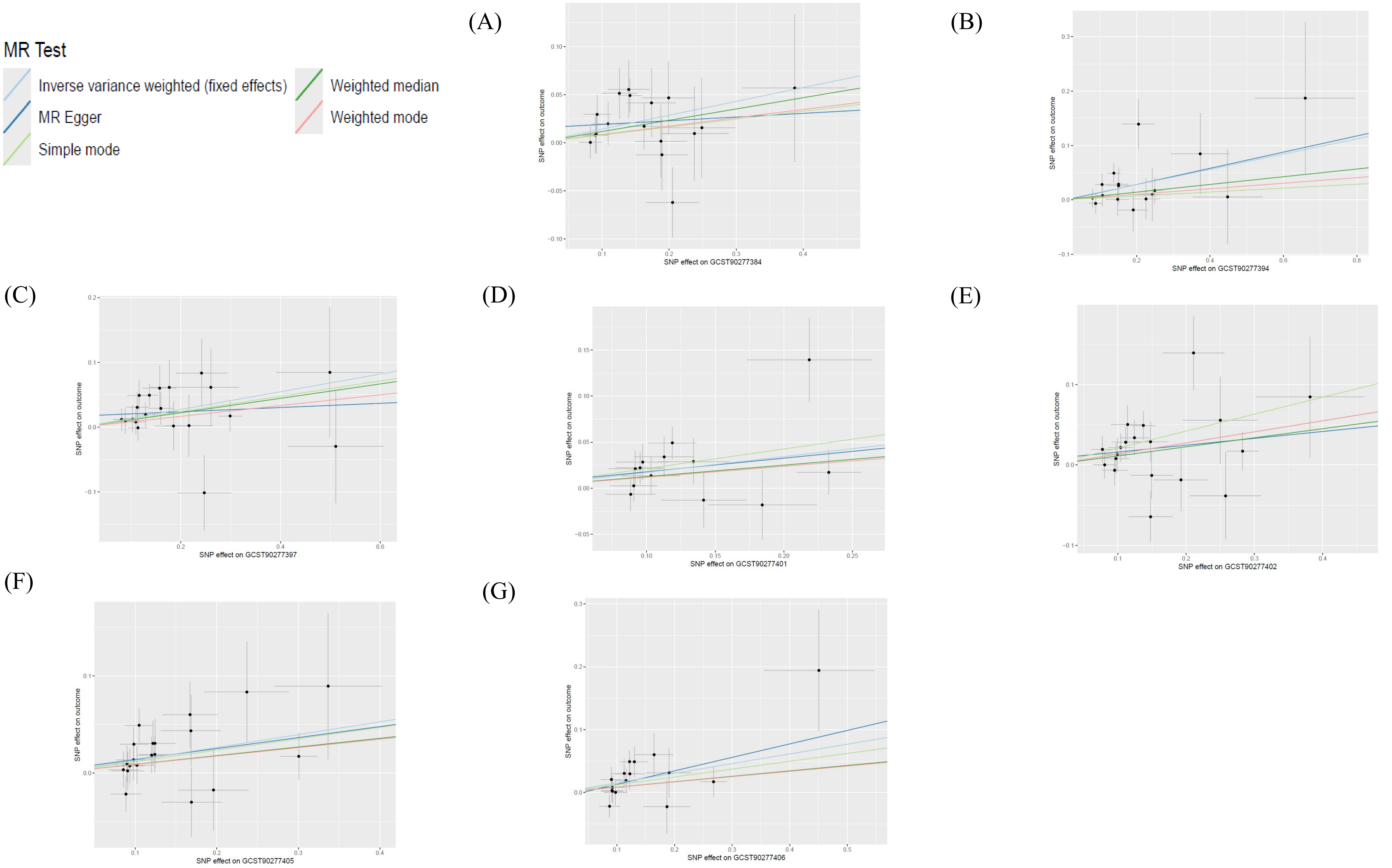

Supplementary Fig. 15. Scatter plots to show MR estimates of (A) TAG(56:3), (B) PC(18:0_0:0), (C) PC(20:4_0:0), (D) PC(14:0_16:0), (E) PC(14:0_18:1), (F) PC(15:0_18:2) and (G) PC(16:0_16:0) on migraine without aura (MO).

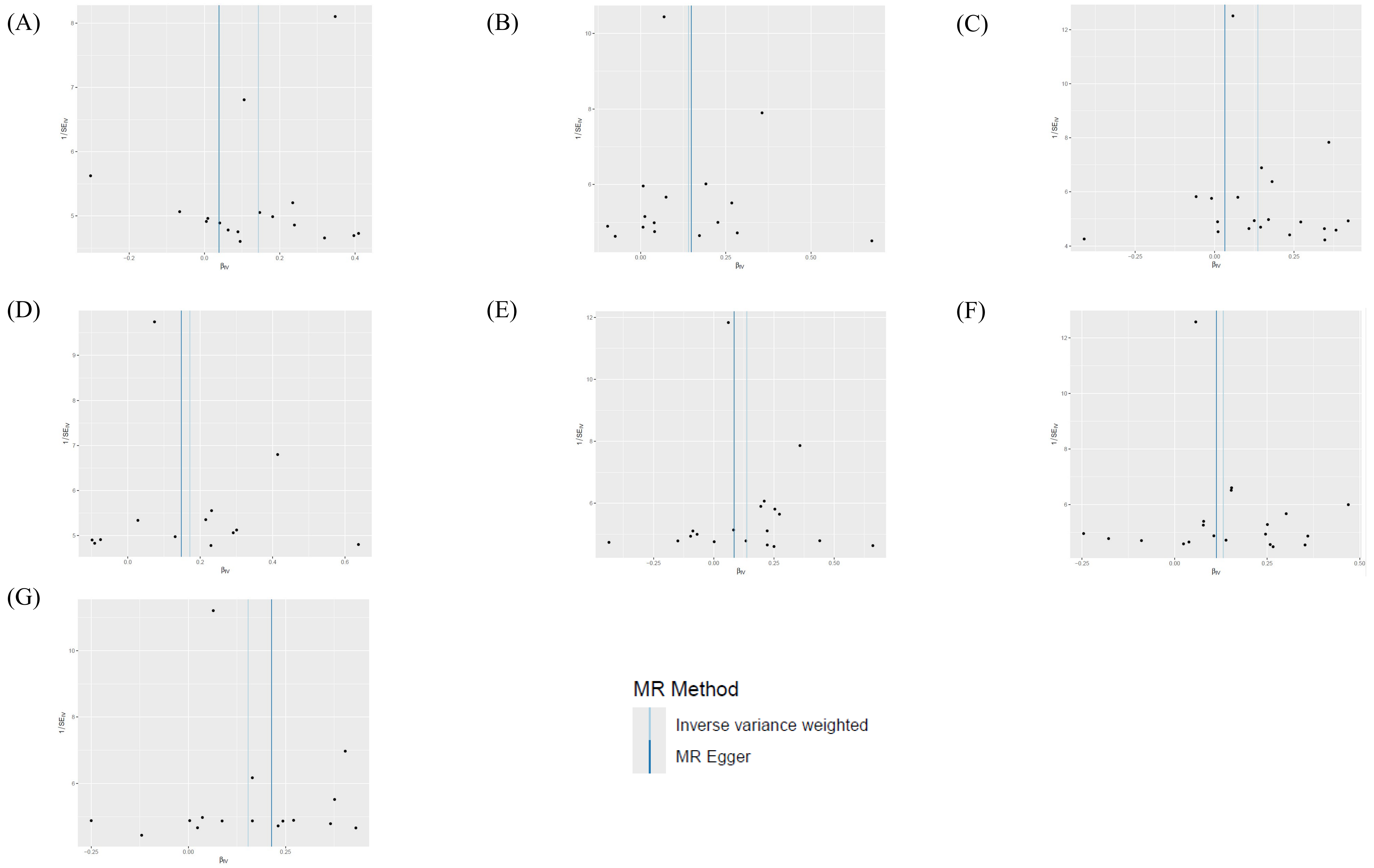

Supplementary Fig. 16. Funnel plots of MR analyses of (A) TAG(56:3), (B) PC(18:0_0:0), (C) PC(20:4_0:0), (D) PC(14:0_16:0), (E) PC(14:0_18:1), (F) PC(15:0_18:2) and (G) PC(16:0_16:0) on migraine without aura (MO).

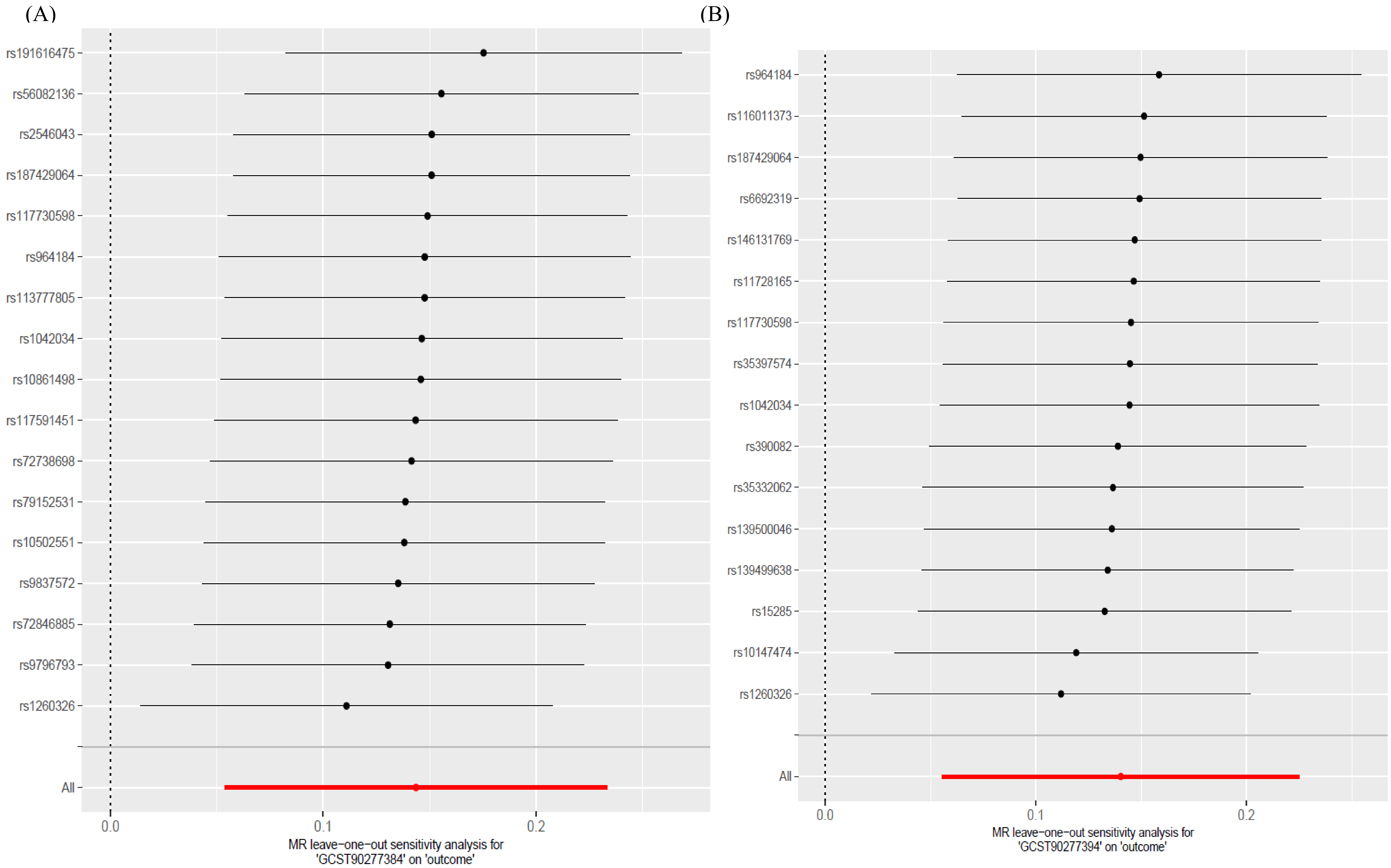

(A)
rs191616475
rs56082136
rs2546043
rs187429064
rs117730598
rs964184
rs113777805
rs1042034
rs10861498
rs117591451
rs72738698
rs79152531
rs10502551
rs9837572
rs72846885
rs9796793
rs1260326
All
0.0
0.1
0.2
MR leave−one−out sensitivity analysis for
'GCST90277384' on 'outcome'
(B)
rs964184
rs116011373
rs187429064
rs6692319
rs146131769
rs11728165
rs117730598
rs35397574
rs1042034
rs390082
rs35332062
rs139500046
rs139499638
rs15285
rs10147474
rs1260326
All
0.0
0.1
0.2
MR leave−one−out sensitivity analysis for
'GCST90277394' on 'outcome'

(C)

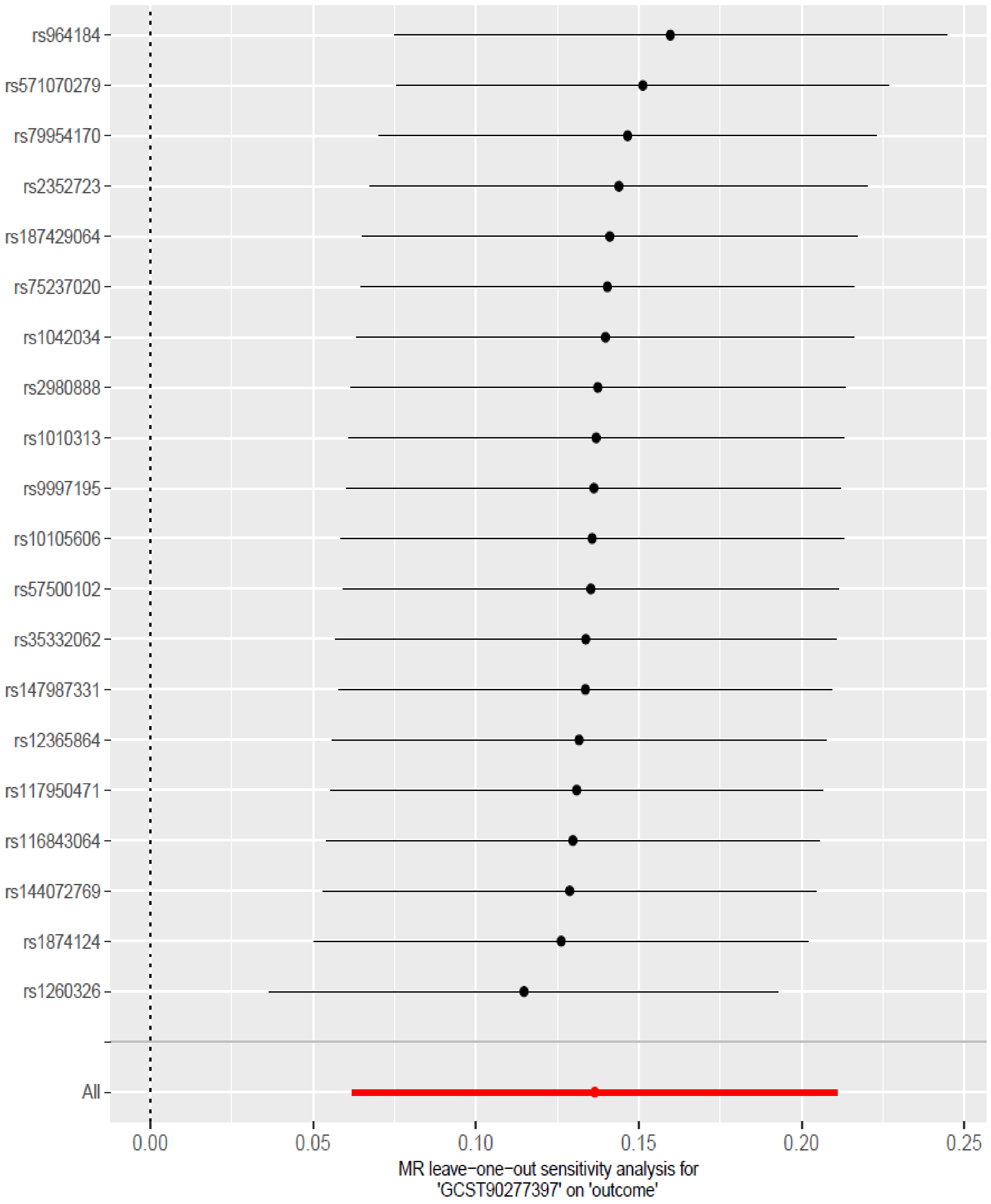
rs964184
rs571070279
rs79954170
rs2352723
rs187429064
rs75237020
rs1042034
rs2980888
rs1010313
rs9997195
rs10105606
rs57500102
rs35332062
rs147987331
rs12365864
rs117950471
rs116843064
rs144072769
rs1874124
rs1260326
All
0.00
0.05
0.10
0.15
0.20
0.25
MR leave−one−out sensitivity analysis for
'GCST90277397' on 'outcome'

(D)

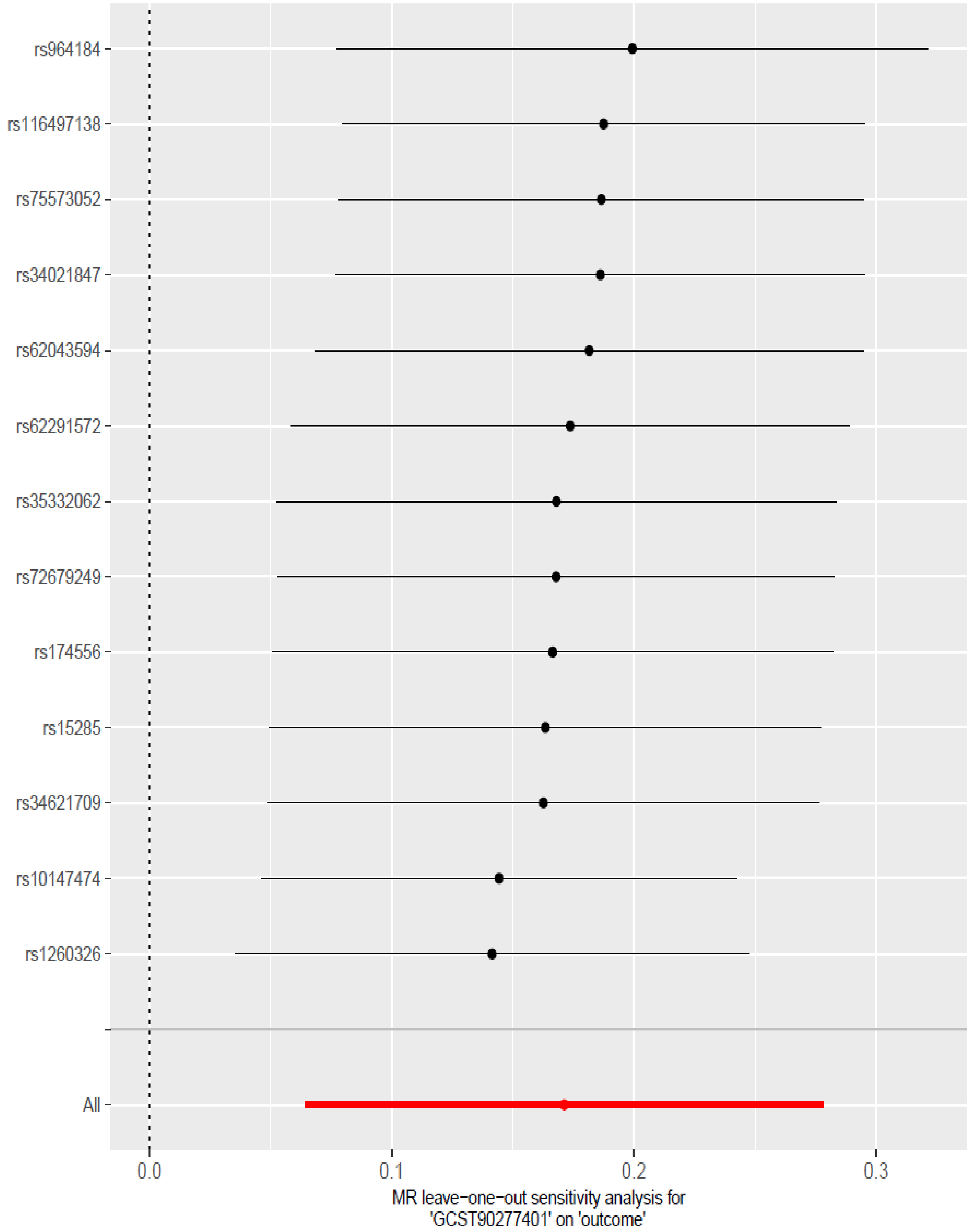
rs964184
rs116497138
rs75573052
rs34021847
rs62043594
rs62291572
rs35332062
rs72679249
rs174556
rs15285
rs34621709
rs10147474
rs1260326
All
0.0
0.1
0.2
0.3
MR leave−one−out sensitivity analysis for
'GCST90277401' on 'outcome'

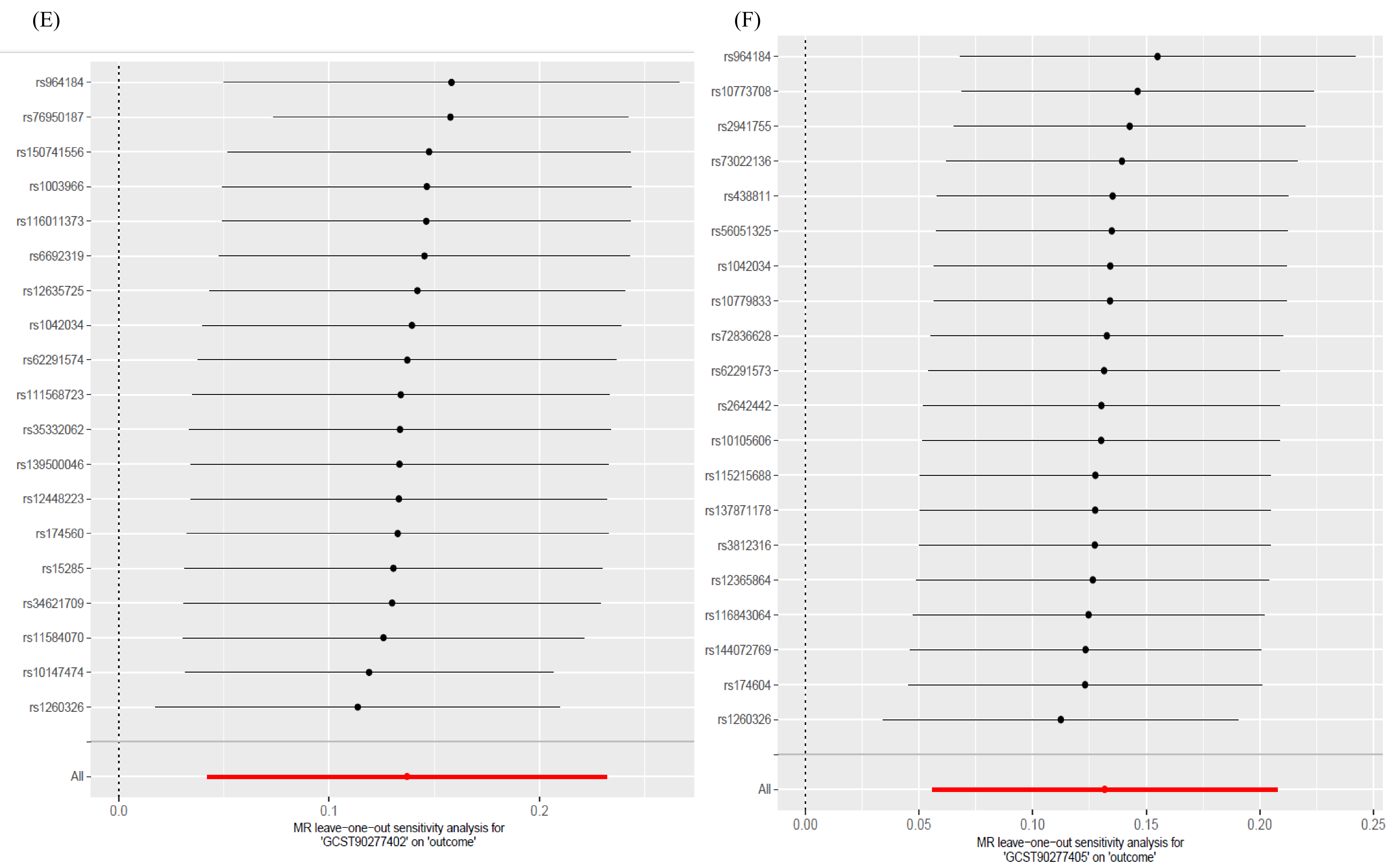
(E)
rs964184
rs76950187
rs150741556
rs1003966
rs116011373
rs6692319
rs12635725
rs1042034
rs62291574
rs111568723
rs35332062
rs139500046
rs12448223
rs174560
rs15285
rs34621709
rs11584070
rs10147474
rs1260326
All
0.0
0.1
0.2
MR leave−one−out sensitivity analysis for
'GCST90277402' on 'outcome'
(F)
rs964184
rs10773708
rs2941755
rs73022136
rs438811
rs56051325
rs1042034
rs10779833
rs72836628
rs62291573
rs2642442
rs10105606
rs115215688
rs137871178
rs3812316
rs12365864
rs116843064
rs144072769
rs174604
rs1260326
All
0.00
0.05
0.10
0.15
0.20
0.25
MR leave−one−out sensitivity analysis for
'GCST90277405' on 'outcome'

(G)

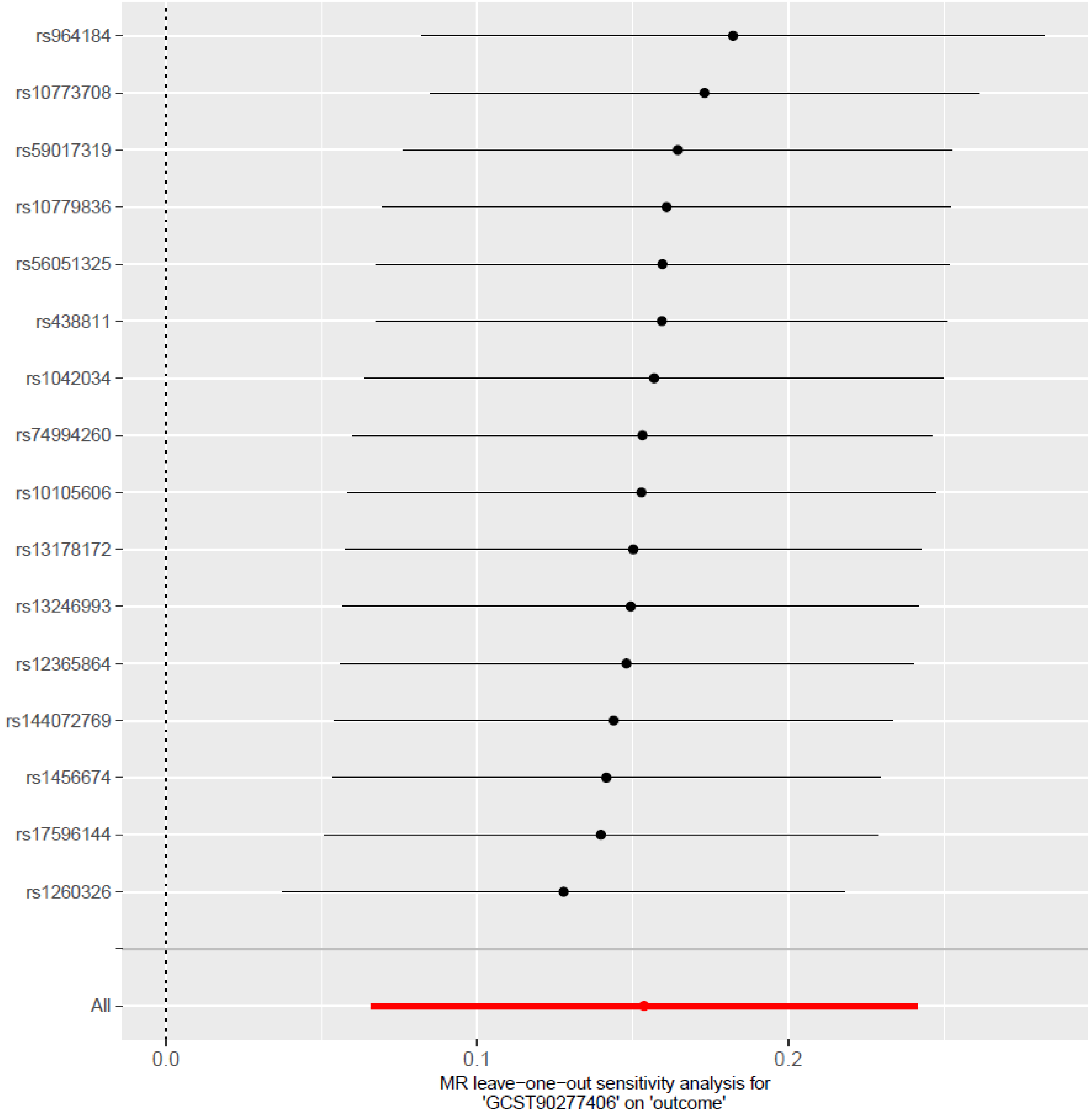

Supplementary Fig. 17. Leave-one-out analysis of MR analyses of (A) TAG(56:3), (B) PC(18:0_0:0), (C) PC(20:4_0:0), (D) PC(14:0_16:0), (E) PC(14:0_18:1), (F) PC(15:0_18:2) and (G) PC(16:0_16:0) on migraine without aura (MO).

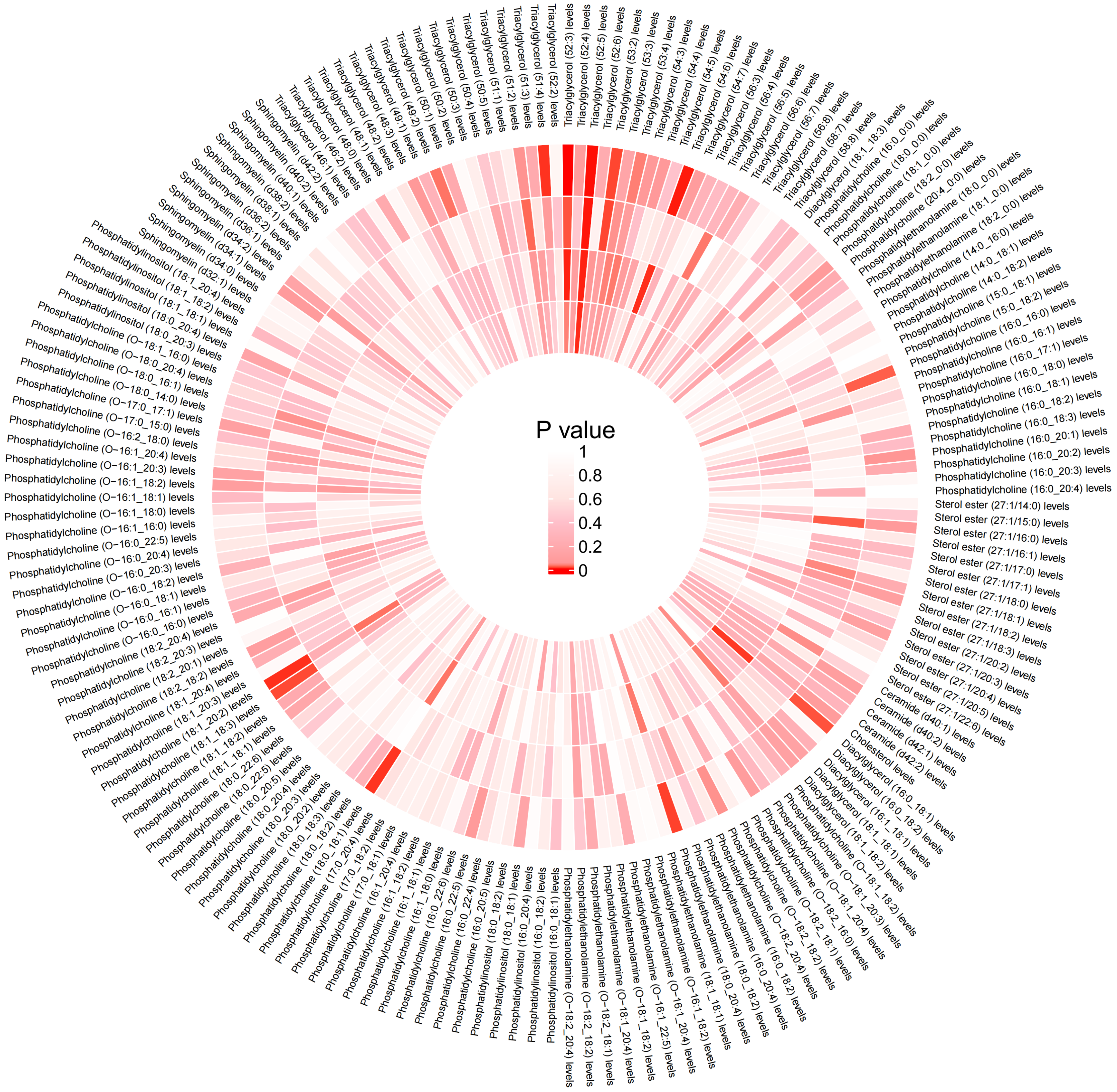

Supplementary Fig. 18. Circular heatmap of the causal effect of 179 lipid species components on migraine with aura. The circular heat map represents the four MR methods, including IVW, Weighted Median, MR-Egger, Weighted mode, in order from the outer ring to the inner ring.

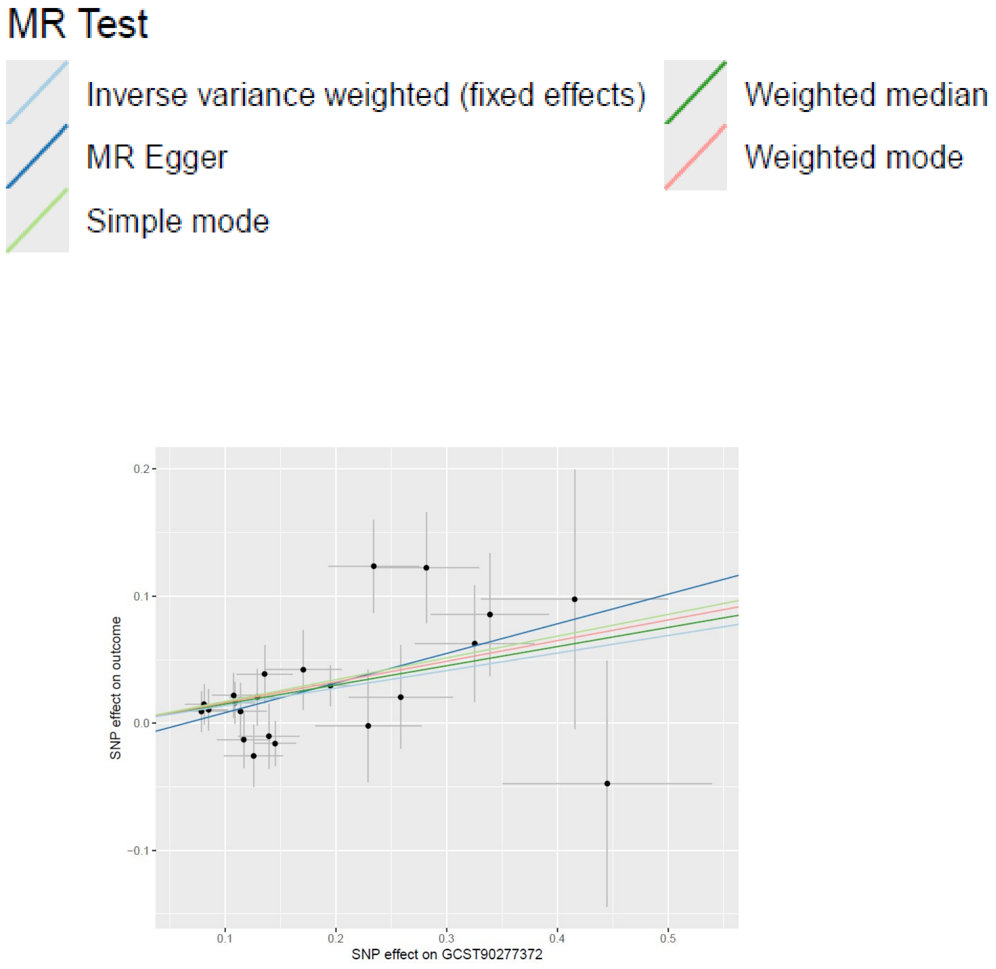

Supplementary Fig. 19. Scatter plots to show MR estimates of TAG(52:3) on migraine with aura (MA).

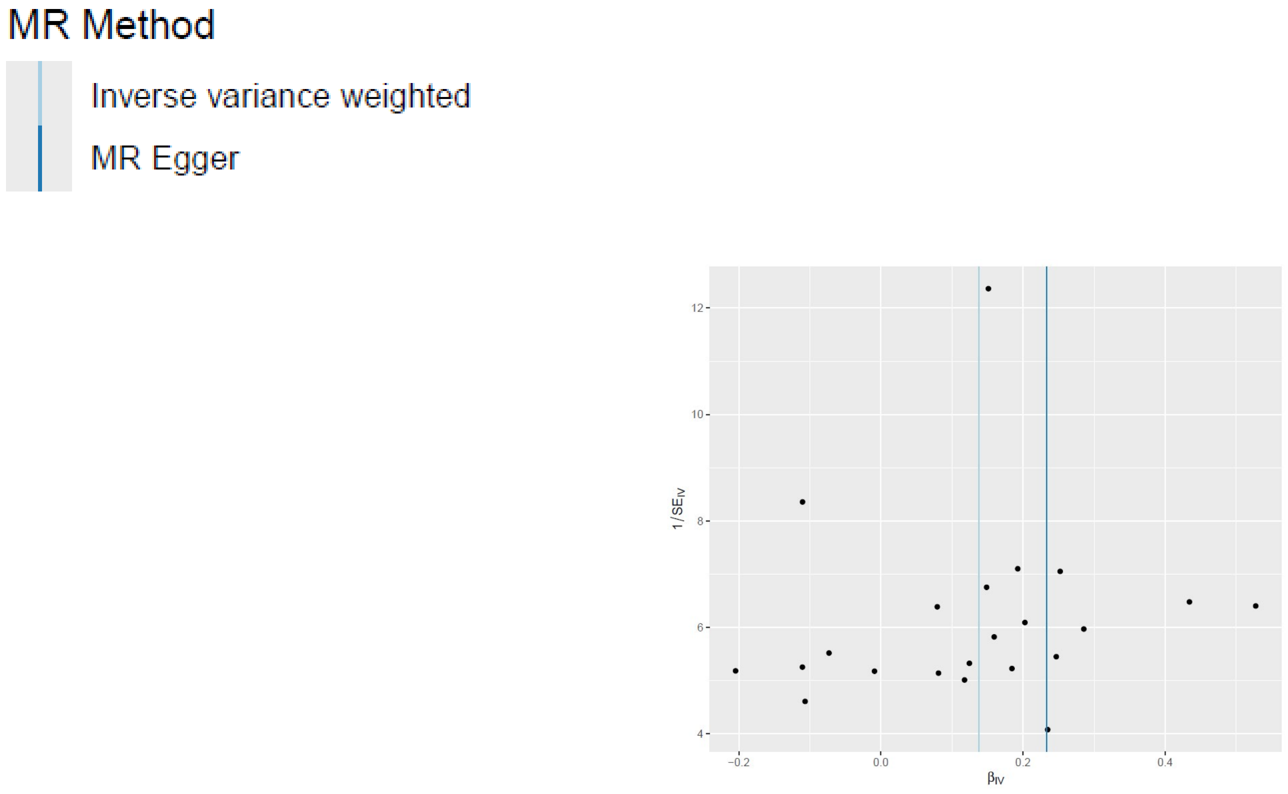

Supplementary Fig. 20. Funnel plots of MR analyses of TAG(52:3) on migraine with aura (MA).

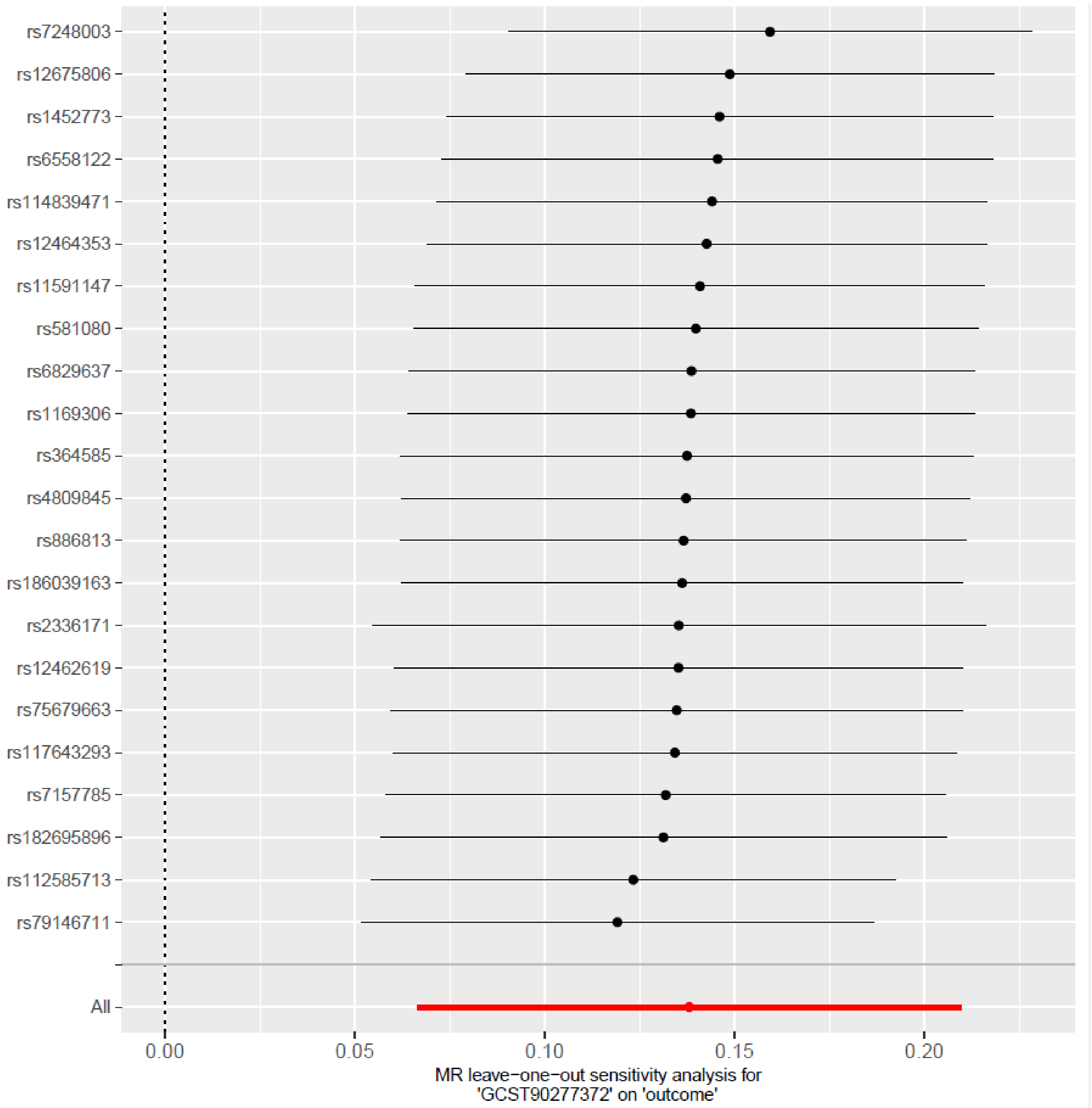

Supplementary Fig. 21. Leave-one-out analysis of MR analyses of TAG(52:3) on migraine with aura (MA).
